# Microclimate investigation of vehicular traffic on the urban heat island through IoT-Based device

**DOI:** 10.1016/j.heliyon.2022.e11739

**Published:** 2022-11-17

**Authors:** Emir Husni, Galang Adira Prayoga, Josua Dion Tamba, Yulia Retnowati, Fachri Imam Fauzandi, Rahadian Yusuf, Bernardo Nugroho Yahya

**Affiliations:** a School of Electrical and Information Engineering, Institut Teknologi Bandung, Bandung, CO, 40132, Indonesia; b Industrial and Management Engineering, Hankuk University of Foreign Studies Yongin, Gyeonggi, CO, 17035, South Korea

**Keywords:** Urban heat island, (UHI), Theoretical UHI, (*UHI*_*T*_), Anthropogenic Heat Flux, queue rate model, fix station sensor

## Abstract

Anthropogenic is defined as one of the influencing factors of the climatic phenomenon, called Urban Heat Island (UHI), in which urban areas have higher air temperatures than their rural surroundings. Analyzing the impact of anthropogenic factors, such as vehicular traffic, has implications for the potential benefits of health monitoring systems; however, the spatiotemporal impact of anthropogenic factors, as well as vehicle mobility, has not been thoroughly investigated. This study incorporates vehicle mobility data by leveraging two different sensors; fixed station sensor instruments designed to integrate with the Internet of Things (IoT), and the camera of an area traffic control system (ATCS) that uses CCTV visualization with object detection. Using object detection and time-based traffic volume analysis, we obtain the level of the queue rate (λ) to present the level of the traffic flow based on the average velocity of the vehicle flow. Based on the results, it showed that the average temperature in urban areas is higher than in suburban areas, and the severe traffic jams caused a significant increase in temperature, that is until 7 Celsius when the weather is sunny. In addition, the theoretical UHI (*UHI*_*T*_) model developed in this study can be used to estimate the UHI which is influenced by the queue rate.

## 1 Introduction

Anthropogenic is a term relating to the influence of human activities on nature. The negative effects of human activities cause some problems such as air pollution. Anthropogenic air pollution is one of the biggest causes of air pollution problems (K. Wang et al., 2021) in addition to natural pollution, namely volcanic eruptions, forest fires, biotic decomposition (Mariano and Christianini, 2021), dust, plant spores, and so on. Air pollution has an impact on a more global problem, namely climate change, with a different contrast situation. For example, a day may include hot weather followed by a sudden and swift rain (Irwansyah, 2016). The extreme impact of climate change causes various problems including mass death disasters (Battacharya, 2003), partial disturbances (Han et al., 2021), premature mortality (Khomenko et al., 2021), and social and economic problems (Fisher et al., 2021). The most detrimental anthropogenic sources are generally associated with the combustion process of various types of fuels, for example, industrial activities and vehicular traffic. Industrial activities as a stationary source with equipment and facilities, such as power generation, automotive production, food processing, clothing, and other production (S. Wu et al., 2021), contribute greatly to climate change. Vehicular traffic as a non-stationary source, such as public and private transportation, contributes to gas emissions (Yu et al., 2021). The problem of climate change caused by anthropogenic is a problem that requires control from various parties, both researchers and the government as regulators. In the future, the need for energy will increase (Handayani et al., 2017) while the source of fossil fuels will decrease (Bebbington et al., 2020) (Bilgen, 2014). Slowly, the automotive industry began to shift towards electric cars which would require lithium, nickel and others as raw materials and may cause other environmental problems such as extreme climate change (X.Wu et al., 2021). Consequently, a rigorous study on urban heat islands (UHI) is required to anticipate aforementioned issue.

The most common approach to investigate UHI is to use remote sensing (Kang et al., 2020) (Habibie et al., 2021). As a tropical country, Indonesia is different from mid-latitudes countries such as the United States, Europe, and Australia, with no heat waves occurring in land rich areas (Meehl and Tebaldi, 2004) (Tomczyk and Bednorz, 2016) (Añel et al., 2017). In fact, the use of remote sensing for UHI observation is limited by the finite spectral bandwidth and the significant out-of-band response from the sensor. Due to the increase in vehicular traffic at many road points, observing the UHI at a particular time point remains a challenge (Louiza et al., 2015). Furthermore, there has been little research work using fixed weather stations to investigate hyperlocal road weather data.

To address the mentioned problems, this study aims to use fixed weather stations to investigate the microclimate UHI. It leverages Internet-of-Things (IoT) to collect the temperature data and camera to detect vehicle to measure the queue rate estimation. The queue rate estimation along with temperature data combined with the weather data will be the input for anthropogenic analysis. The analysis takes place not only to investigate the extreme UHI but also to estimate the queue rate in vehicular traffic and the anthropogenic heat flux to construct a vehicle velocity model and UHI theoretic model. Our contribution to this study can be described as follows:

• To leverage IoT to collect temperature data for UHI measurement
• To propose Theoretical UHI (*UHI*_*T*_) to empirically measure the UHI as the alternative of UHI Measurement (*UHI*_*M*_)
• To test the significancy of the proposed Theoretical UHI (*UHI*_*T*_) with the UHI Measurement (*UHI*_*M*_)

The experiment has been conducted on the local road located in Bandung City, which has a population of 2,5 million and is noted as the 14^th^ most congested city in Asia (Asian Development Bank, 2019). The city of Bandung is the largest metropolitan city in the West Java province, and the third largest city in Indonesia. In terms of population density, Bandung is the second most populous city in Indonesia after Jakarta, with an average population of approximately 15,000 people per square km. Based on these facts, it is important to analyze the phenomenon of microclimate and UHI in Bandung city.

The remaining sections of this paper are organized to coherently address the objectives of this research. The related works which discuss similar issues related to UHI are described in section 2. In section 3, the methodologies for urban temperature measurement and vehicle counting for vehicular traffic analysis are describe. Section 4 addresses the results of UHI observations and the vehicular traffic analysis. Section 5 concludes the study.

## 2 Literature review

UHI refers to a climatic phenomenon in which an urban area or a metropolitan area is significantly warmer than the surrounding suburban areas due to human activities such as vehicular traffic (Louiza et al., 2015). There are several causes of UHI, such as dark road surfaces that absorb significantly more solar radiation, geometric effects of tall buildings, the car mobility policy that effects caused many issues, such as the spectacular growth of fuel consumption, etc (Sun et al., 2019).

Generally, there are two approaches to capture UHI phenomenon (Bahi et al., 2020) (Kong et al., 2021). The first is remote sensing, which uses satellites to get the surface temperature. This method has high spatial coverage but lacks temporal resolution. The second method is in-situ measurement, which aims to directly measure the air temperature. The method consists of two different ways, i.e., mobile transect, and fixed station and they are often used together. Mobile transect moves at predetermined hours and lines. It requires a large workforce to obtain high spatial coverage. Fixed stations attempt to measure at a particular observation point from time to time and require a lot of sensor placement to get high spatial coverage. By implementing the Fix station system, it can work in a continuous way and is compact sized to allow flexible placements in various locations. Fix station system can reliably be developed and deployed into a consumer product for use as an UHI monitoring device with high accuracy. Other than that, fix station system could work to collect, publish, subscribe, and store data automatically, it is still necessary to further improve the system for its reliability (Fauzandi et al., 2021).

A study on UHI using remote sensing method investigated the effect of urban heat anomaly on the risk of heat-related mortality and the association of various land use and land cover (LULC) indicators with urban heat anomaly and heat-related mortality (Jang et al., 2020). They proved that the high urban heat anomaly correlates to low vegetation and high urban surface indicators and the urban heat anomaly was positively associated with the heat-related mortality risk. Other research work utilizes remote sensing to identify specific regions in phoenix that have been increasingly heat-ed and cooled to further understand how LULC change influences the surface urban heat island intensity (C. Wang et al., 2016). They proved that the regions that experienced the most significant land surface temperature change during the study period are primarily on the outskirts of the Phoenix metropolitan area for both daytime and nighttime.

The alternative measurement is to use fixed stations, such as the application of a low-cost measurement network in Bern, Switzerland (Gubler et al., 2021) by considering a low-cost measurement device (LCD) consisting of a temperature logger and a custom-made, naturally ventilated radiation shield. It had been used to inter-comparison with automated weather stations (AWS) at three reference sites during the record-dry summer of 2018. Other research compared between mobile transect and fix stations for the applicability to represent the microclimate condition in urban areas (Shi et al., 2021). However, among the existing studies, the spatiotemporal impact of anthropogenic activities along with vehicle mobility has not been thoroughly investigated.

Decreased air quality and warm temperatures are the effects of the queue rate in urban traffic. By measuring the vehicle traffic density, it is able to find the correlation on the microclimate phenomenon, similar to the macroscopic or microscopic traffic analysis method that describe the traffic stream with a series of parameters (Bazzan and Klügl, 2013) Vehicle velocity (*v*) is considered as a microscopic property of each vehicle. Hence, the average speed of vehicle traffic can be associated with the whole stream. The macroscopic parameters are mostly related to the traffic volume (traffic flow, flow rate), and the density. Density-macroscopic measure of the vehicle proximity is defined as the number of vehicles occupying a given length of a road or lane, expressed as vehicles per length unit. Macroscopic parameters can be obtained from microscopic ones (such as vehicle positions and speeds) by local aggregation (Michrandi Nasution et al., 2019).

Study about anthropogenic heat flux (AHF) estimation such as (Xu et al., 2021) (Wang et al., 2020) (Bonifacio-Bautista et al., 2022) (Chen et al., 2020) (Wu et al., 2022) (Mei and Yuan, 2021) (Alhazmi et al., 2022) provide an overview of the method for estimating the AHF value. The article (Xu et al., 2021) conducted a study to obtain a model of the dynamic AHF estimation value approach by integrating multisource Internet big data and high-precision urban spatial data (Xu et al., 2021). applies satellite remote sensing, urban spatial modeling, UAV monitoring, and data mining (Xu et al., 2021). assume that the urban AHF is considered to buildings, traffic, and human metabolism (Xu et al., 2021). claim that the AHE changed violently from $498\;W.m^{-2}$ to above $1000\;W.m^{-2}$ in central areas where land use functions are to a high degree blended and urban forms and are complicated. The results of their investigation showed the emission intensity of various land properties showed obvious differences, and commercial land had the largest average AHF values followed by residential, industrial, education, others, and green land. On the other hand, their AHF (Wang et al., 2020) study claims constructing the AHF estimation model with good accuracy and time-variation consistency. Research data (Wang et al., 2020) is obtained from inventory as secondary data. Research (Bonifacio-Bautista et al., 2022) explains how to estimate the main sources of AHF, based on a vehicle classification (taxis, cars, minibuses and buses), surveys of electricity consumption and population density in Mexico City. They claim that the highest heat emission occurs from 15:00 to 21:00 h. AHF generation in dense areas of Mexico City, can exceed $75\;W.m^{-2}$. According to (Bonifacio-Bautista et al., 2022) vehicle flow observations based on the busiest intersection at each site resulted in a large heat flux vehicle (*Qv*) (Chen et al., 2020). research gives the view that a reliable and accurate representation of AHF is still lacking. Therefore, need a machine learning-based top-down approach to generate a gridded AHF benchmark dataset at 1 km spatial resolution (Chen et al., 2020). uses secondary data in conducting the research process. According to (Chen et al., 2020) AHF values in urban centers of metropolises range from $60\;to\;190\;W.m^{-2}$ and highest AHF value up to $415\;W.m^{-2}$ in heavy industrial zones (Wu et al., 2022). stated that the components of AHF, including their calculation methods and temporal characteristics, have not been investigated. In the AHF calculation process (Wu et al., 2022) approaches the heat transfer coefficient and uses inventory data. They claim that the maximum values of AH at 12:00 were $35,15\;W.m^{-2}$. Based on the actual studies related to AHF that have been done that there are several things that need to be done to improve the accuracy of AHF measurements in order to get more reliable results for specific areas. Like the research conducted by (Wu et al., 2022) (Bonifacio-Bautista et al., 2022) applying a good approach, but improvements need to be made, such as making a computer vision model capable of counting vehicles to counting people with high accuracy (accuracy >90%). Furthermore, by modeling the approach to the physics of heat mass transfer and its mathematical phenomena. Last but not least is Convenient to get complete weather data at an affordable.

## 3 Methodology

### 3.1 Research framework

**Figure 1:**
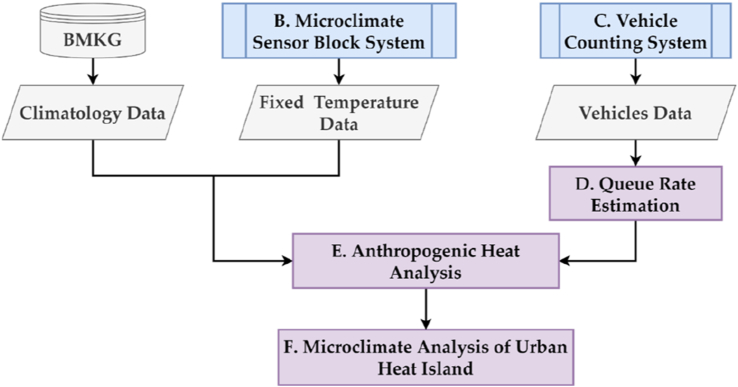
Microclimate investigation framework.

This section aims to discuss the research framework on this study (Figure 1). The microclimate investigation framework starts with temperature data collection and vehicle counting data system. The temperature data collection is a primary data that is obtained from the microclimate sensor. The microclimate sensor block system is a mechanism to get fixed temperature data. The temperature data is combined with the climatology data, obtained from the meteorological agency called BMKG (Badan Meteorologi, Klimatologi, dan Geofisika). The vehicle counting system leverages camera to count the vehicles. This study developed vehicle object detection based on computer vision method to count the number of vehicles. The result of the vehicle counting system is a queue rate estimation is combined with the temperature and climatology data to result anthropogenic heat flux. At the final stage, microclimate analysis of UHI takes place.

The implementation of sensors in this research is focused on urban areas with environmental parameters of high congestion, high population density, high buildings and high population activity. The population data in the city of Bandung is about 2.5 million people and the annual growth of vehicles is 1 million units (Badan Pusat Statistik Kota, 2020).

### 3.2 Microclimate sensor block system

**Figure 2:**
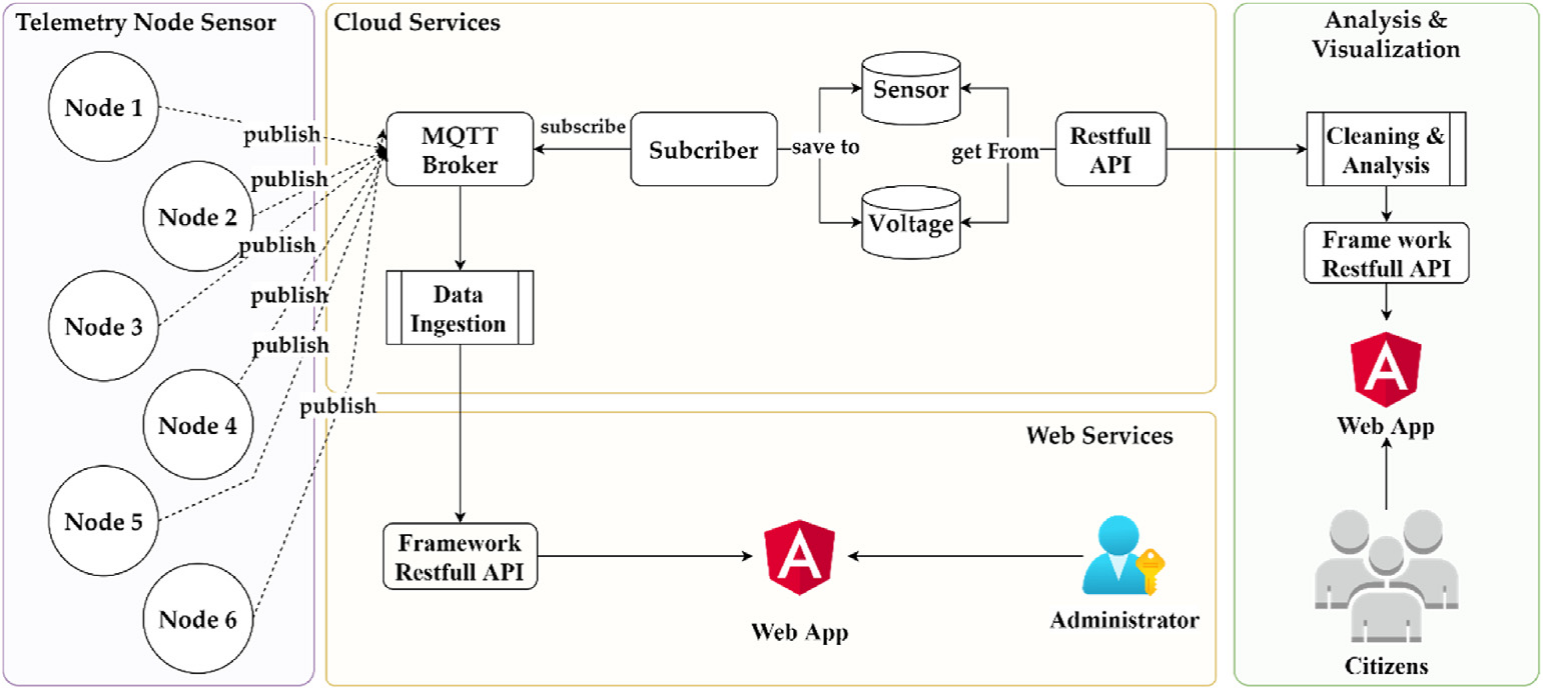
Microclimate sensor block system.

The overall system of microclimate sensor block is shown at Figure 2. It consists of four main services, telemetry node sensor, cloud services, web service and analysis & visualization. Telemetry node sensor should be flexible and easy to install at the observation site, e.g., by attaching the sensor to a pole. Cloud services take on the role as data aggregators and provide access to the data for further analysis. Web service is provided to facilitate the registering telemetry node sensor to be installed at the observation area and to provide detailed information about telemetry node sensor. The analytical tools are using Flask and Google colab to make it easier for user to analyze UHI data and its correlation with anthropogenic heat parameters. Flask is a Python web framework built with a small core and easy-to-extend philosophy to analyze temperature and humidity based on historical data using Python.

The telemetry node sensor is encapsulated in an enclosure box. A Stevenson screen is utilized to cover the tip of the sensor to avoid direct solar radiation (Fauzandi et al., 2021). explains how the sensor collects data at every defined timestamp and sends data every “N”-th minutes of an hour. DIY board sensor utilizes Arduino ProMini 8Mhz as a controller to provide low power, and sensor module using SHT31 with temperature and humidity accuracy of ±0.2 °C and ±2 %RH, respectively (Sensirion, 2016). The board contains an RTC (Real-Time Clock) DS3231 to maintain a wake-up signal and an AT24C32 EEPROM (Electrically Erasable Programmable Read-only Memory) to store the data temporarily. The communication module implements SIM800L over a 3rd Generation Network. The temperature sensor is placed on the road shoulder with a 2-meter radius from the road. To avoid vandalism the sensor is installed at an altitude between 3 and 4.5 m above ground level (Figure 4), which is above the standard height of 2 m but within the acceptable range for urban canopy layer temperature measurements.

**Figure 3:**
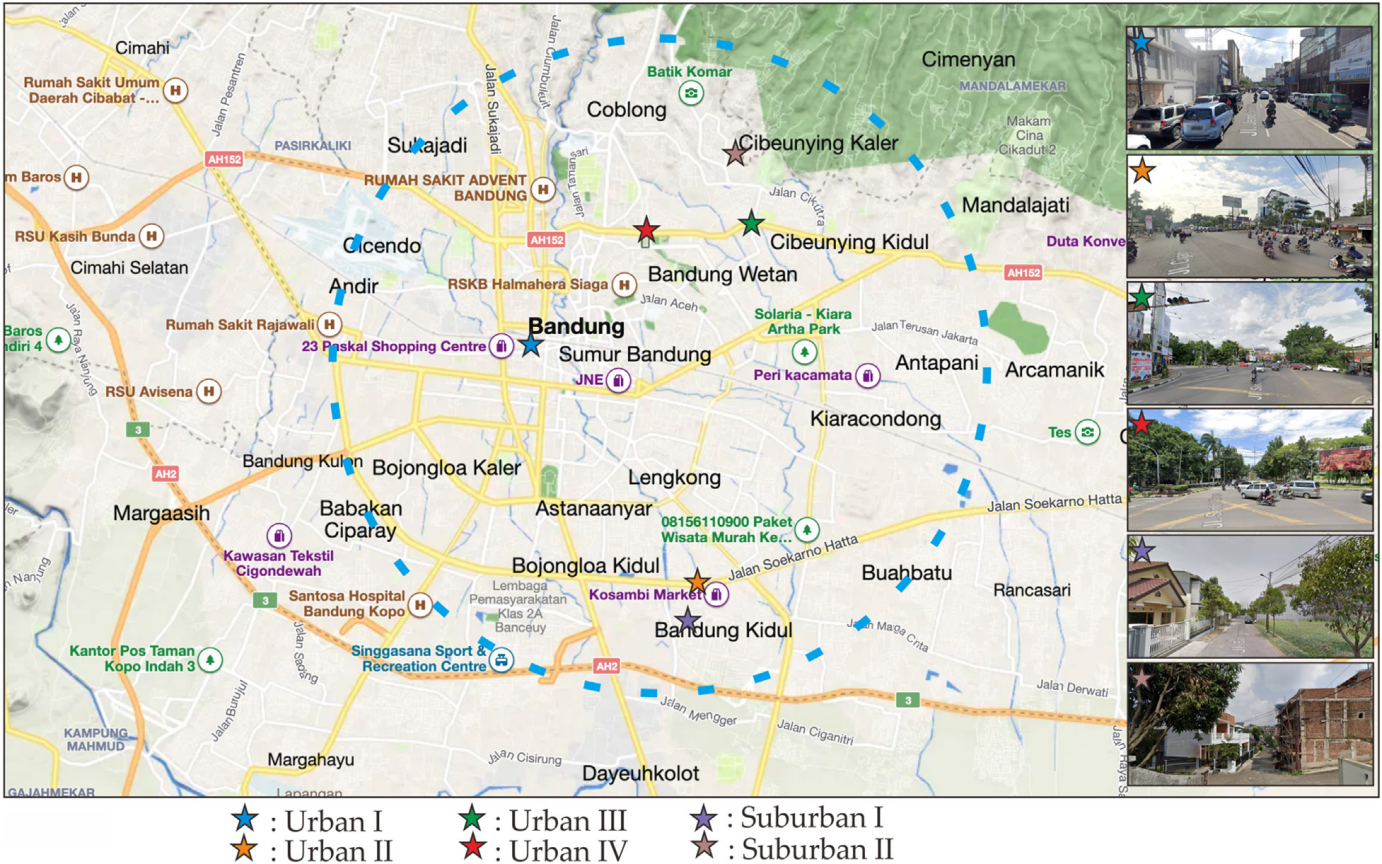
Geospatial of urban and suburban research area in bandung Indonesia.

**Figure 4:**
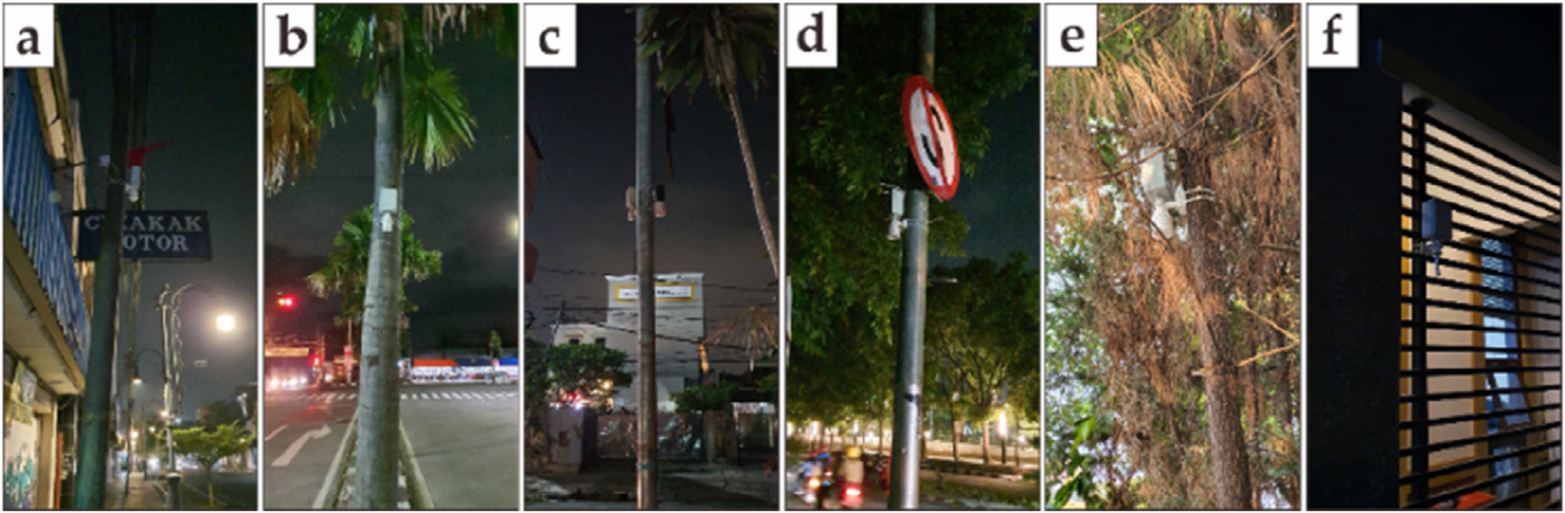
Sensor implementation.

In this study, we placed sensors in six (6) locations consisting of four (4) urban areas and two (2) suburban areas (Figure 3). Urban area I is a trading area with very dense buildings and very little vegetation (Ruas Otista Street, Figure 4a). Urban Area II is the southern ring road of the city of Bandung which connects the eastern and western areas of the city of Bandung. In addition, in Urban Area II, the condition of the building is dense and there is little vegetation (Sp. Batununggal, Figure 4b). Urban Areas III and IV are the northern ring roads of the city of Bandung which connect the western and eastern areas of the city of Bandung. In addition, in urban areas III (Sp. Pahlawan, Figure 4c) and urban areas IV (Sp. Telkom, Figure 4d), there are dense buildings and sufficient vegetation. Suburban areas I (Batununggal residence, Figure 4e) and suburban areas II (Cikutra residence, Figure 4f) are residential areas with sufficient vegetation and dense buildings. The detailed location is shown in Table 1.

**Table 1:** Location of Fix Station sensor placement.

Index	Location	Street	Latitude	Longitude
Node 1 (a)	Urban I	Ruas Otista	-6.920615	107.602744
Node 2 (b)	Urban II	Sp. Batununggal	-6.949885	107.626101
Node 3 (c)	Urban III	Sp. Pahlawan	-6.897758	107.634188
Node 4 (d)	Urban IV	Sp. Telkom	-6.899292	107.619276
Node 5 (e)	Suburban I	Batununggal residence	-6.954817	107.623313
Node 6 (f)	Suburban II	Cikutra residence	-6.888136	107.633219

Cloud services aim to collect and store data. Referring to (Fauzandi et al., 2021), all cloud service components utilize Amazon Web Services. The telemetry node sensor communication uses Mosquito as an open-source MQTT Broker (Massage Queuing Telemetry Protocol) in a Virtual Machine from AWS EC2. The subscriber also uses tools on AWS EC2, runs NodeJS, and stores data in AWS DynamoDB. Finally, the JSON Rest API is used to provide data access for further analytics in the analytical tool, that is Google Colab and Python Flask. Web service and analytical tool will be discussed in the result section.

### 3.3 Vehicle counting system

Vehicle type tracking and classification has emerged in the domain of traffic management (Sarikan et al., 2017) (H. Wang et al., 2018). There are two methods for carrying out the vehicle detection process: traditional methods and deep learning-based methods (Uçar et al., 2017). Currently, vehicle detection objects using a convolutional neural network (CNN) has a higher performance than the traditional method (Ghrabat et al., 2019) (Zhang et al., 2019). One of the leading approaches to object detection with CNNs method is the YOLO framework (Redmon et al., 2016). This study utilizes YOLO as an object detection framework due to its reliability and applicability.

**Figure 5:**
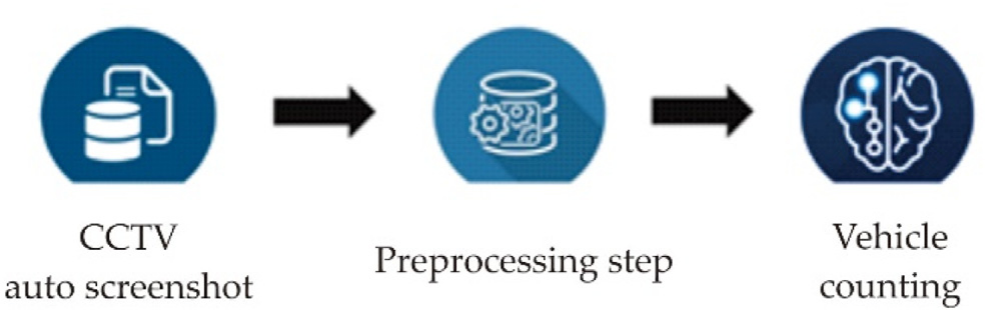
Vehicle counting system architecture.

The data source is CCTV (Closed circuit television) that had been installed in some area in Bandung city. Each frame of video obtained from CCTV is stored every second using a Python script and the vehicle counting process is carried out separately for resource efficient utilization. The data collection process utilizes the Raspberry Pi and the vehicle counting process is carried out on another device using YOLOv5. The vehicle detection and the counting result is exported into a CSV file. The overall flow of the vehicle counting system consists of three (3) parts, CCTV Auto Screenshot, Cleaning Data, and Vehicle Counting. The illustration for this system can be seen in Figure 5.

The system works in the three sequential steps: CCTV auto screenshot, preprocessing, and vehicle counting. The *CCTV auto screenshot step* captures a frame of CCTV video every second. One of the functions on this capturing process is to store the data on a Raspberry Pi. Each captured frame is saved in a JPG file format. The *preprocessing step* is to reformat and cut the image in accordance with the desired conditions after the auto-screenshot process within a specified time. The *vehicle counting step* is to count the number of vehicles after the data is preprocessed. This process begins with object detection using the YOLOv5 framework. The result of the object detection step is a value that represents the number of vehicles and is subsequently exported to a CSV file.

CCTV auto-screenshot is a process of capturing an image from a desired CCTV and exporting the relevant data from the image every second. The data source is obtained from CCTV called SP Batunuggal and SP Otista which can be accessed via the link http://atcs-dishub.bandung.go.id/. This video data format is m3u8. The auto-screenshot step starts by feeding the video into a system that contains a Python script to read and convert it into a JPG image format. The OpenCV library is used to cut the required frames from the video. The results of each captured frame will be exported to a jpg format file. This process is executed on the Raspberry Pi with the pm2 process manager to keep the script running.

Before doing the vehicle calculation process, the image should be cleaned so that unnecessary information will not be processed. The preprocessing step is carried out automatically using a Python script. This script contains a noise removal technique to remove unnecessary parts of the image by cropping function. The script uses the OpenCV library for image editing. For the resource efficiency, we split the process between the clearning data and auto screenshot process. Images that are already stored on the Raspberry Pi will be downloaded periodically, in this case, daily.

We carry out the vehicle counting process automatically using YOLOv5 framework. This algorithm has many advantages over deep learning target detection framework. YOLOv5 provides several models that can be used to perform object detection. This study utilizes YOLOv5s as a model to carry out the vehicle detection process because of its superior speed in detecting vehicles. The results of the YOLOv5 process will be further processed using a Python script to obtain the number of existing vehicles. This script will also export the results to a csv file.

**Figure 6:**
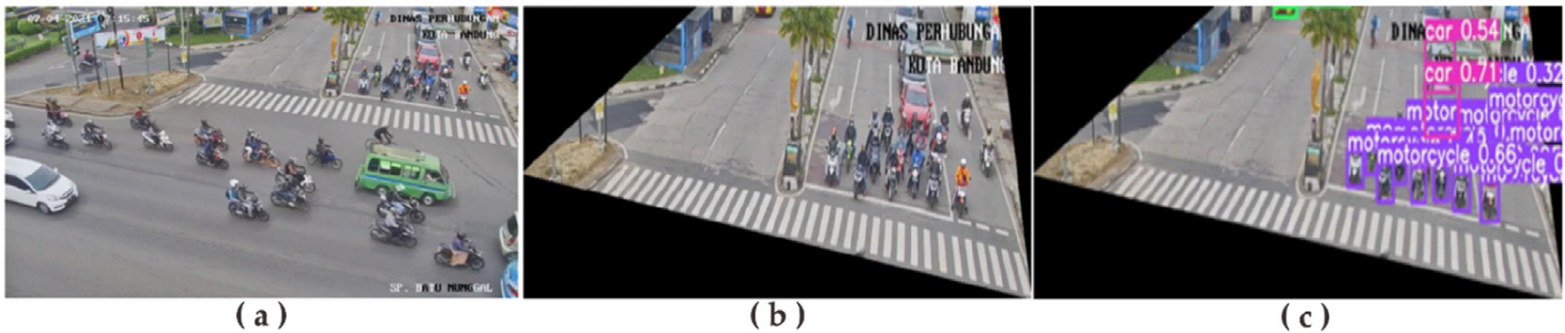
Sampling data (a) before (b) after cleaning data (c) Object detection process.

System testing begins with testing the CCTV auto screenshot process that has been in-stalled on the Raspberry Pi. This test was carried out for seven (7) days with a timer set every second. From test results, a script that has been set and activated can take pictures for seven (7) days every 2 s. Furthermore, a testing data cleaning process is carried out. Testing is done by cleaning data in the image that has been taken in the previous process. Judging from one of the results of data cleaning, Figure 6a shows the process before data cleaning and Figure 6b shows the process after data cleaning. For image cropping conditions in this test, the image is taken only at the top intersection.

Next, images that have gone through the data cleaning process will be processed using YOLOv5 to obtain vehicle detection results. From these results, this system can perform a vehicle detection process for almost all vehicles in the image. Vehicles that are not detected in the image are caused by being covered by other vehicles in this image. Another factor from other test results shows that a vehicle that is not detected is caused by lighting and blur. After entering the cleaning process, a vehicle counting process will be carried out. The results of this process can be seen in Figure 6c. Finally, we tested the performance of this system by doing a comparison between the number of real vehicles (*RC)* and the results of this system (*SC)*. The percentage of accuracy is calculated using Eq. (1) (Fachrie, 2020).

(1) $$Accuracy=\left(1-\frac{\left|RC-SC\right|}{RC}\right)\times 100\%$$

### 3.4 Queue Rate estimation

The queue rate estimation measure takes into account traffic flow (*q*), average velocity (*v*), and the number of vehicles (*N*). All these measurements are calculated as an average. The queue rate (*λ)* is a value that represents the level of traffic congestion. The higher the rate value, the tighter the congestion. The traffic density of vehicle ($\sigma_{v}$) parameter can be calculated by the average speed of passing vehicles and average number of vehicles per minute. As Little's Theorem explains in the concept of queuing theory, the number of vehicle (*N*) in a stable-state queuing system is related to the queue rate (*λ)* and the time required in system (*T)*. Mathematically, it can be written as Eq. (2) below:

(2) N=λ·T

**Figure 7:**
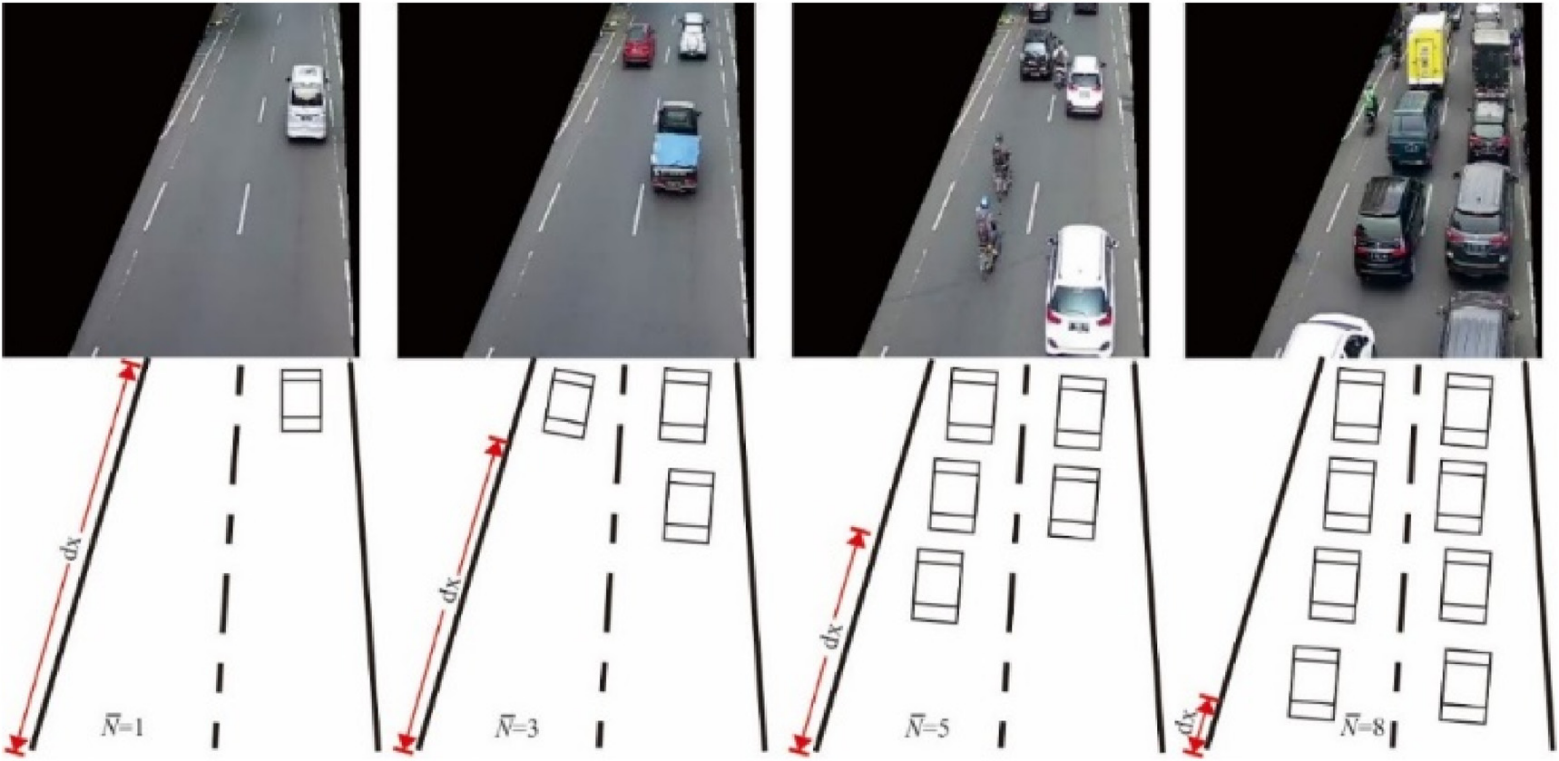
Queue rate (*λ)* 30–450 (unit/minute).

In the problem of traffic jams, we assume that on the condition of the freeway (λ = 0) the average vehicle speed is 80 km/h. If the queue rate increases, $\left(\lambda_{i}>\lambda_{0}\right)$ then the vehicle speed will decrease $\left(v_{i}<v_{0}\right)$ and is also influenced by the length of the track (*x*) (Figure 7) and a constant (*k*). Therefore, mathematically it can be written as Eq. (3) below:

(3) $$\lambda =\frac{N}{T}=-\frac{dv}{k\cdot dx}$$

Vehicles running into a decrease in velocity (*dv*) will have a deceleration (*a*). Estimated deceleration experienced by vehicles as a result of queue rate (*λ*), which can be written as Eq. (4) below:

$$a=\frac{dv}{dt}=\left(\frac{dx}{dt}\right)\cdot \frac{dv}{dx}=v\cdot \frac{dv}{dx}$$

(4) $$a=v\cdot \frac{dv}{dx}=v\cdot \left(-k\lambda \right)=-k\lambda v$$

Then the estimated vehicle speed (*v*) which is influenced by the queue rate (*λ*) is:

$$\frac{dv}{dt}=-k\lambda v$$

$$\frac{dv}{v}=-k\lambda dt$$

$$\ln\;{v|}_{v_{0}}^{v}=-k\lambda t$$

(5) $$v=v_{0\;}e^{-k\lambda t}$$

**Figure 8:**
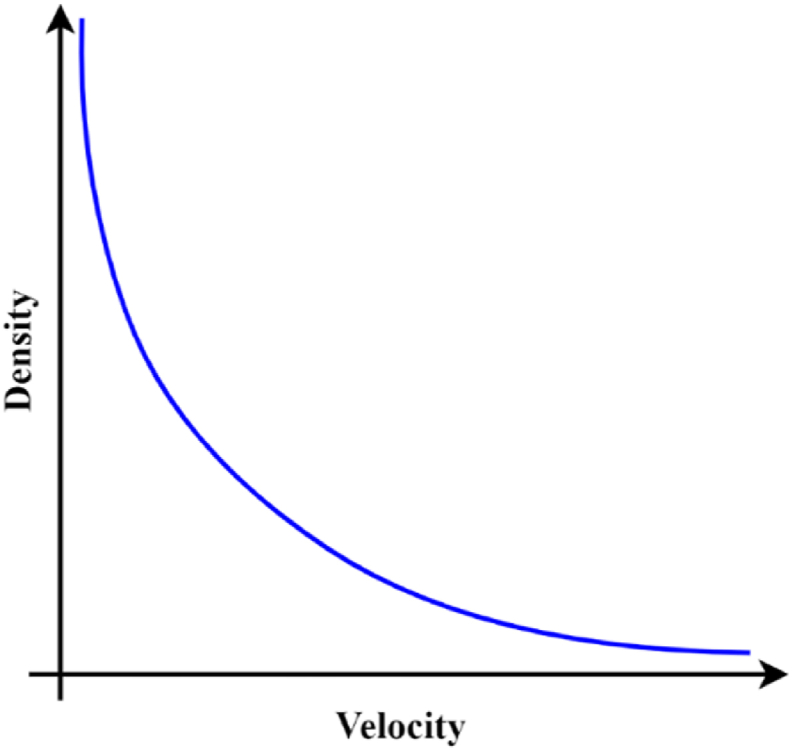
Fundamental diagram of traffic parameters (Bazzan and Klügl, 2013).

Eq. (5) is a vehicle velocity model that is affected by the queue rate (*λ*). Theoretically, the correlations between flow (i.e., velocity and density) are illustrated in Figure 8. The higher the flow caused a higher density until the road could not accommodate other vehicles. When there are lots of vehicles on the road, the velocity of each vehicle would be lower than the average.

### 3.5 Anthropogenic heat analysis

In this section, we develop a theoretical UHI as an empirical UHI instead of tedious tasks on measuring the UHI. The theoretical UHI (${UHI}_{T}$) is the measurement result to see the difference between surrounding suburban areas and the urban areas with a consideration of the heat coefficient. The UHI measurement (${UHI}_{M}$), which is the measurement result from the sensors, can be calculated based on time intervals. UHI can be calculated every minute for one full day (24 h) using Eq. (6) (Sun et al., 2019) (Schatz and Kucharik, 2015).

(6) $${UHI}_{M}\;\left({}^{\circ}C\right)=T_{u}-T_{s}$$

Anthropogenic heat flux is phenomenon that appears in cities due to the alteration of energy balance caused by various factors such as vehicle density (queue rate), local climate, energy consumption, heat generate by human metabolism, and so on (Grimmond, 1992). All the various factors that have been mentioned will accumulate to form an anthropogenic heat flux (*Q*_*f*_). There are different methods to estimate *Q*_*f*_*,* such as energy balance residual, inventories and top-down and bottom-up methods. In this study, anthropogenic heat flux (*Q*_*f*_) can be represented as the sum of various factor of the urban environment as shown in Eq. (7) below (Grimmond, 1992).

(7) $$Q_{f}=Q_{V}+Q_{B}+Q_{M}\left[W\cdot m^{-2}\right]$$

where $Q_{V}$ is the heat emitted by internal combustion vehicles, $Q_{B}$ is the heat release mainly by energy consumption by buildings. $Q_{M}$ is the heat generated by human metabolism. The $Q_{V}$ emission can be estimated from Eq. (8) below (Grimmond, 1992):

(8) $$Q_{V}=\left[\sum_{ijk}\left(\lambda_{ijk}\left(h\right)\left({EV}_{ij}\right)d_{k}\right)\right]/A\;\left[W\cdot m^{-2}\right]$$

Where *h* refers to time in hours, A is the heat contribution area, *h* is the time in hour and $\lambda_{ijk}$ is the total number of vehicle per hour of *i* class vehicles that consume fuel type *j* and moving in the route segment *k*, $d_{k}$ is the distance in which the vehicle is transported in the segment *k* (m) and ${EV}_{ij}$ is the energy used by the vehicle class *i* which consumes the fuel type *j*, given by Eq. (9) below:

(9) $${EV}_{ij}=\left[NHC_{j}\left(p_{j}\right)\right]/FE_{ij}\;\left[J\cdot m^{-1}\right]$$

Where $NHC_{j}$ is the combustion of net heat of the fuel type *j* (J · kg^−1^); *p*_*j*_ is the density of the fuel type *j* (kg · L^−1^); *FE*_*ij*_ is the average fuel economy of class vehicles *i* that consume the type of fuel *j* (m · L^−1^). In physical terms, the ${UHI}_{T}$ equation can be formulated with the following Eq. (10) below:

(10) $${UHI}_{T}\;\left({}^{\circ}C\right)=\frac{\;Q_{fu}-Q_{fs}}{\bar{h}}$$

Where, $Q_{fu}$ is the heat flux in urban area, $Q_{fs}$ is the heat flux in suburban area, and $\bar{h}$ is heat transfer coefficient. The $\bar{h}$ depends on several factors including the length of the medium through which the fluid flows, the conduction heat transfer coefficient of the medium, the velocity of the fluid flow and the kinematic viscosity of the fluid (Kothandaraman, 2006). To calculate $\bar{h}$, we use Eq. (11) below:

(11) $$\bar{h}=0.664\times \frac{k}{L}\cdot {Re}_{L}^{0.5}\cdot {\mathrm{Pr}}^{0.33}\left[W\cdot m^{-2}C^{-1}\right]$$

With *k* as the thermal conductivity (W/mK), *L* as the length of the medium (*m*), Re as the Reynolds number, and *Pr* as the Prandtl number. Before calculating $\bar{h}$, it is necessary to calculate the Reynolds number for the fluid (air) flowing over the medium. Reynolds equation is shown in Eq. (12) below (Kothandaraman, 2006):

(12) $$Re=\frac{v\times L}{\mu }$$

Where *v* is the velocity of the fluid (m/s), *L* is the length of the medium (*m*) and μ is the coefficient of viscosity (m^2^/s). Values such as thermal conductivity (*k*), coefficient of viscosity (μ), and Prandtl number (*Pr*) are obtained based on the film temperature which is the average of the medium temperature and air temperature.

### 3.6 Microclimate analysis of urban heat island

The null hypothesis (H0) is satisfied with a statement that there is no effect of queue rate on changes in UHI temperature. On the other hand, the alternative hypothesis (H1) is satisfied if the queue rate of vehicle traffic has an effect on changes in UHI. After analyzing the covariance and variance to test the hypothesis, normal distribution, correlation, principal component analysis (PCA) and principal component regression (PCR) were carried out to understanding the relationship and determine the effect of Queue Rate on UHI.

Correlation is a type of nonexperimental research for understanding the relationship between two quantitative variables. Regression considers how one quantitative variable is influenced by another. In correlation analysis, the two quantitative variables are considered symmetrically. Suppose that for each value of a variable *y* (dependent variable as UHI), another variable *x* (independent variable as Vehicles density) has a probability distribution P(y|x), the probability of *y,* given *x.* The mean value of this distribution, alternatively called the expectation of *y*, given *x,* and written E(y|x), is a function of *x* and is called the regression of *y* on *x* (Lindley, 1990).

**Figure 9:**
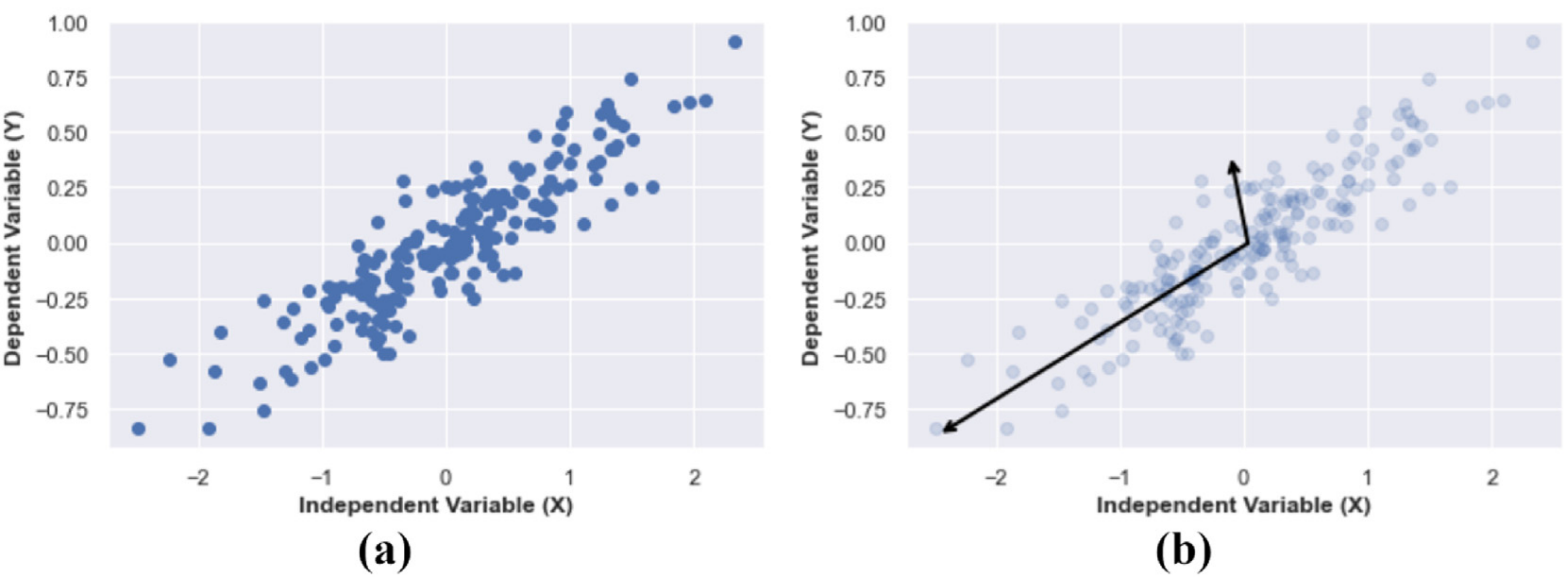
Data random numbers from a variety of probability distributions (a) Scatter Data (b) PCA Data.

PCA is fundamentally a dimensionality reduction algorithm, but it can also be useful as a tool for visualization, for noise filtering, for feature extraction and engineering, and much more (Jollife and Cadima, 2016). After a brief conceptual discussion of the PCA algorithm, we will see how the data of UHI that measure in the urban is correlate with Queue Rate effect. Principle component analysis is a fast and flexible unsupervised method for dimensionality in data, which we saw briefly in introducing Scikit-Learn. Its behavior is easiest to visualize by looking at a two-dimensional dataset. Consider the following data random normalized that consisting variable x and y (Figure 9 a).

In the analysis process using the PCA method, we obtain the eigenvector and eigenvalues from the covariance of the 2-dimensional data. The eigenvector value explains the direction of the vector and the eigenvalue describes the length of the square of the vector value (Figure 9 b). These vectors represent the principal axes of the data, and the length of the vector is an importance indication of that axis in describing the data distribution. In other words, it measures the variance of the data when projected onto that axis. The projection of each data point onto the principal axes are the "principal components" of the data.

Principal component regression (PCR), in high-dimensional setting, the required number of samples may be too great since it scales with the number of features κ, which is large. However, this problem can be circumvented when the covariates have a latent, low-dimensional representation. In particular, PCR (Jolliffe, 1982), has been precisely designed to address such a setting. Using all observed covariates, PCR first finds an r≪κ dimensional representation for each feature using the method of Principal Component Analysis (PCA). Specifically, PCA projects every covariate $A_{i}$, onto the subspace spanned by the top r right singular vectors of the covariate matrix, the concatenation of all observed covariates. PCR then uses the r− dimensional features to perform linear regression. If the covariate matrix is indeed of rank r, then by the theory of Linear Regression, it follows that the number of samples required to achieve vanishing in- and out-of-sample prediction error scales faster than $r\sigma^{2}$, which is significantly smaller when r≪κ. Where the notation of $\sigma^{2}$ is variance (“On Robustness of Principal Component Regression,” 2019).

## 4 Result and discussion

### 4.1 Web service for Microclimate investigation

**Figure 10:**
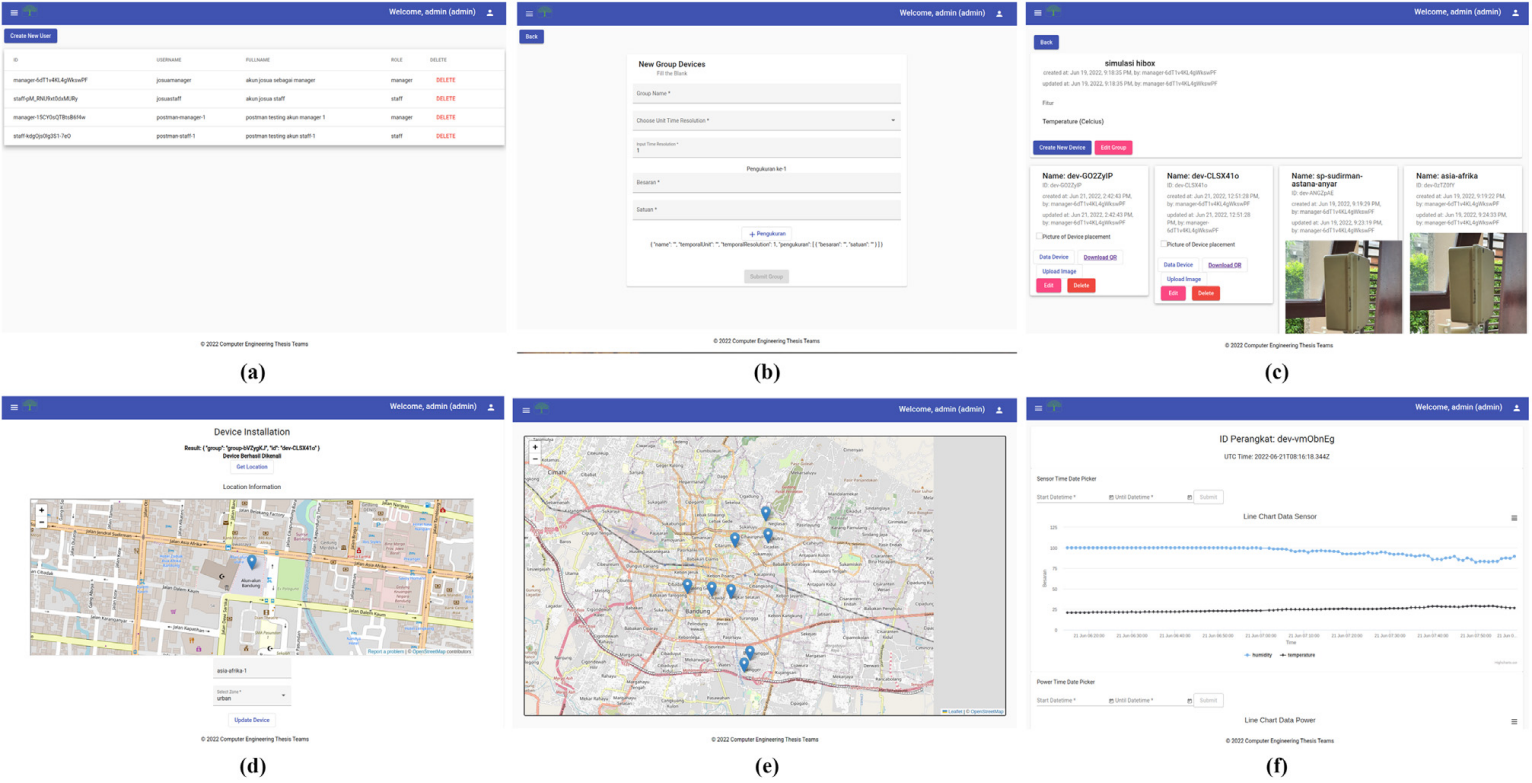
Web Service (a) account list, (b) form to create new group devices, (c) devices on a group, (d) device installation, (e) device installed on map, (f) sensor data visualization from a device.

This section describes web services with various services (Figure 4), such as MQTT broker, database, Restful API, and web application. The MQTT broker is set up to maintain communication from device as the publisher, and the subscriber program to handle incoming data. Measurement results from devices are stored using a reliable Time Series Database (TSDB) for time-based data models. In addition to data from sensors, it is also necessary to store information data about the device itself, such as geolocation and the type of data sent, using a relational database. To interact with data, a Restful API is provided with the HTTP method, in addition to serving data requests on web applications as well, endpoints are also provided for advanced analysis such as integration with the Pandas framework, endpoints require an access token given by the administrator. Finally, to facilitate interaction with data, a GUI is provided using a web application (Figure 10).

Referring to Figure 10, web application has some features such as user management, device information management, and device monitoring. In user management, there are three functions (*user creation*, *user group creation*, and *device registration*. First, *user creation* is a function to create a user account to access this website provided by the admin and this page can only be accessed by admin (Figure 10a). Second, *user group creation* is a function to create a new group manually using the form (Figure 10b). *Device registration* is a function to create a new device and load the device name to the list of devices page. Afterward, the system generates information such as QR Code to help to identify the device and MQTT Topic (Figure 10c). The respective QR code could identify the device by showing the geolocation (Figure 10d). In addition, all the installed devices are mapped into the geolocation (Figure 10e). Subsequently, it is possible to look into the specific device and the data produced by each device is shown on a line chart (Figure 10f).

### 4.2 Urban heat Island (UHI) analysis

**Figure 11:**
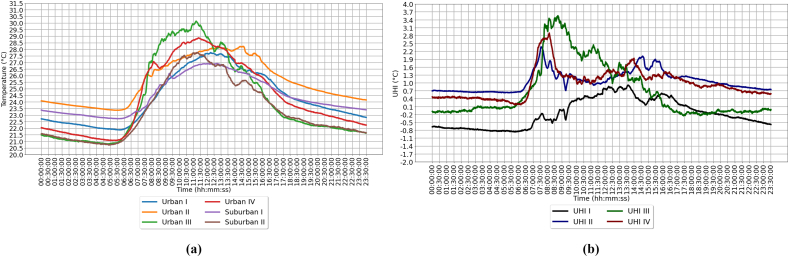
(a) Average air temperature (b) Average UHI during June and November 2021.

The period of the temperature measurements took place between June and November 2021 in the urban and suburban areas of Bandung city. The observation showed that there is a large temperature difference at certain times. In general, the increase in temperature occurs at 06:00–14:00 and the decrease in temperature begins at 14:00–05:00 in sunny weather conditions (Figure 11 a). The condition of this temperature difference is hypothetically influenced by several factors, such as queue rate (λ) traffic jam, lack of vegetation, and weather conditions.

In general, the weather is sunny and cloudy, with the highest average temperature difference of 3.6 Celsius (Figure 11b). On average, throughout June–November, the air temperature in Urban III is the highest compared to the others. As a result, it is possible to conclude that the effect of congestion and queue rate causes an increase in ambient air temperature. Further analysis shows that traffic congestion with an average velocity of 7 km/h causes an increase in air pollution and of course the air quality is very bad. The long-term effects will cause many health problems, such as respiratory diseases, etc.

### 4.3 Result of object detection and Queue Rate estimation

In the process of performance testing, we tested 50 images randomly from the results obtained. The results of the testing process can be seen in Table 2.

**Table 2:** Result of testing performance.

Vehicle	Counting		Counting Error	Overall Accuracy
	Real (RC)	System (SC)		
Car	230	180	-39	79.62%
Motorcycle	340	250	-71	

**Figure 12:**
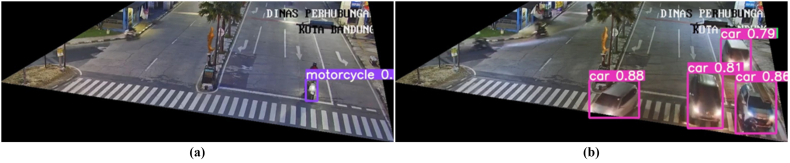
Noise when vehicle counting process (a) blocked by other vehicles (b) blurred image.

From the results of performance tests, there are errors in the results of the vehicle counting process. The biggest error occurs in the number of motorcycles. This is because most of the vehicles, especially motorcycles, are jostled and blocked by other vehicles (Figure 12a). In addition, there are also reading errors due to blurred image conditions and existing lighting conditions (Figure 12b).

**Figure 13:**
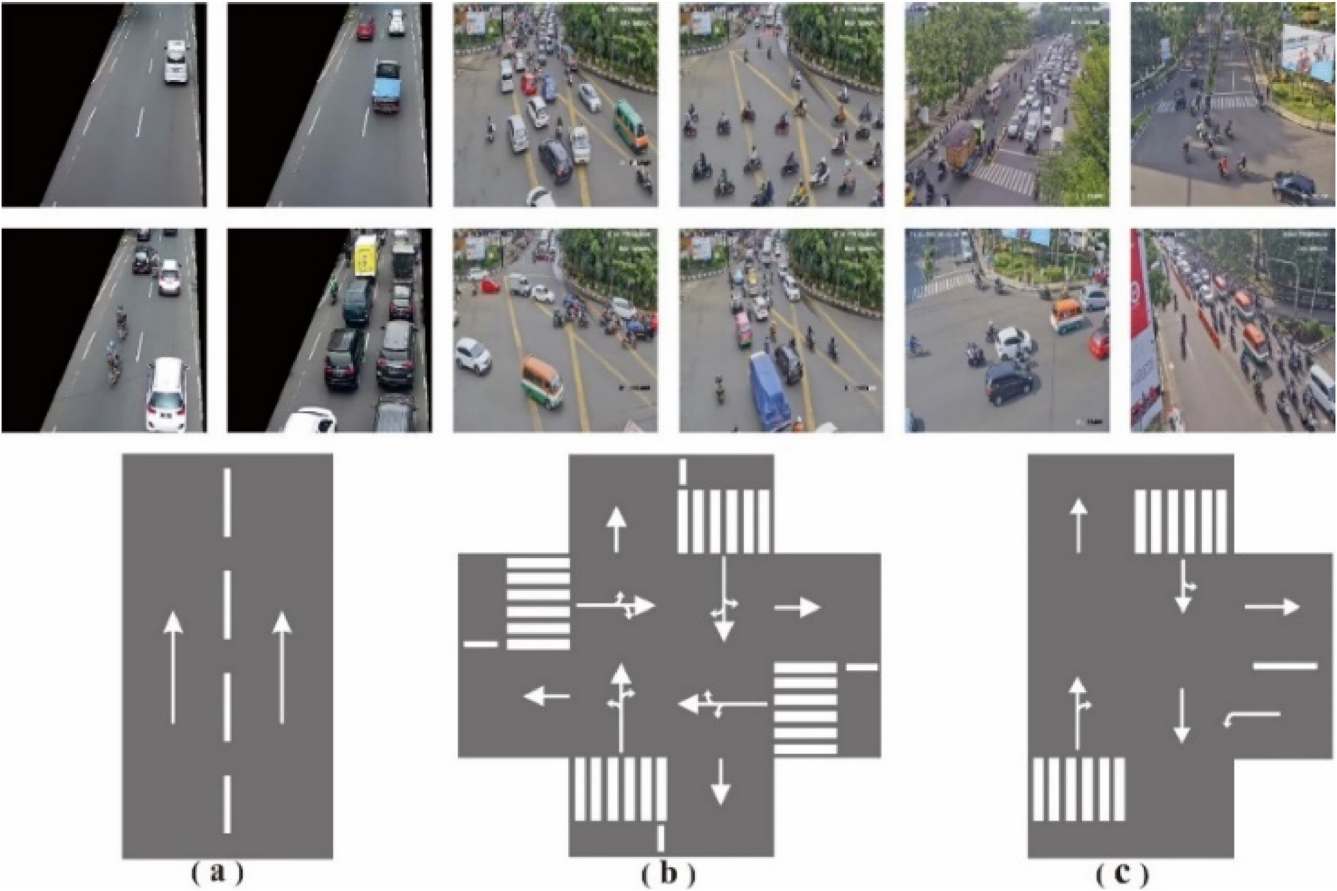
Different model of street (a) Urban I, (b) Urban III and (c) Urban IV.

The queue rate (*λ*) data obtained by the object detection method using the YOLOv5 framework carried out on the Urban I, Urban IV, and Urban III in the city of Bandung Indonesia. The detailed data can be seen in Table 3. Each street has a type of intersection itself. Urban I street has a type of one-way (Figure 13a), Urban IV has three intersections (Figure 13b), and Urban III has four intersections (Figure 13c). Based on the vehicle velocity equation model that is caused by the queue rate (Equation 5), there is a constant (*k*) which is the value obtained based on the type of intersection. While time (*t*) used for 1 min.

**Table 3:** Location of ATCS's CCTV used in this research.

Location	Street	Latitude	Longitude	Additional
Urban I	Ruas Otista	-6.920615	107.602744	https://45.118.114.26/camera/Ruasotista.m3u8
Urban III	Sp. Pahlawan	-6.897758	107.634188	https://45.118.114.26/camera/Pahlawan.m3u8
Urban IV	Sp. Telkom	-6.899292	107.619276	https://45.118.114.26/camera/Telkom.m3u8

**Figure 14:**
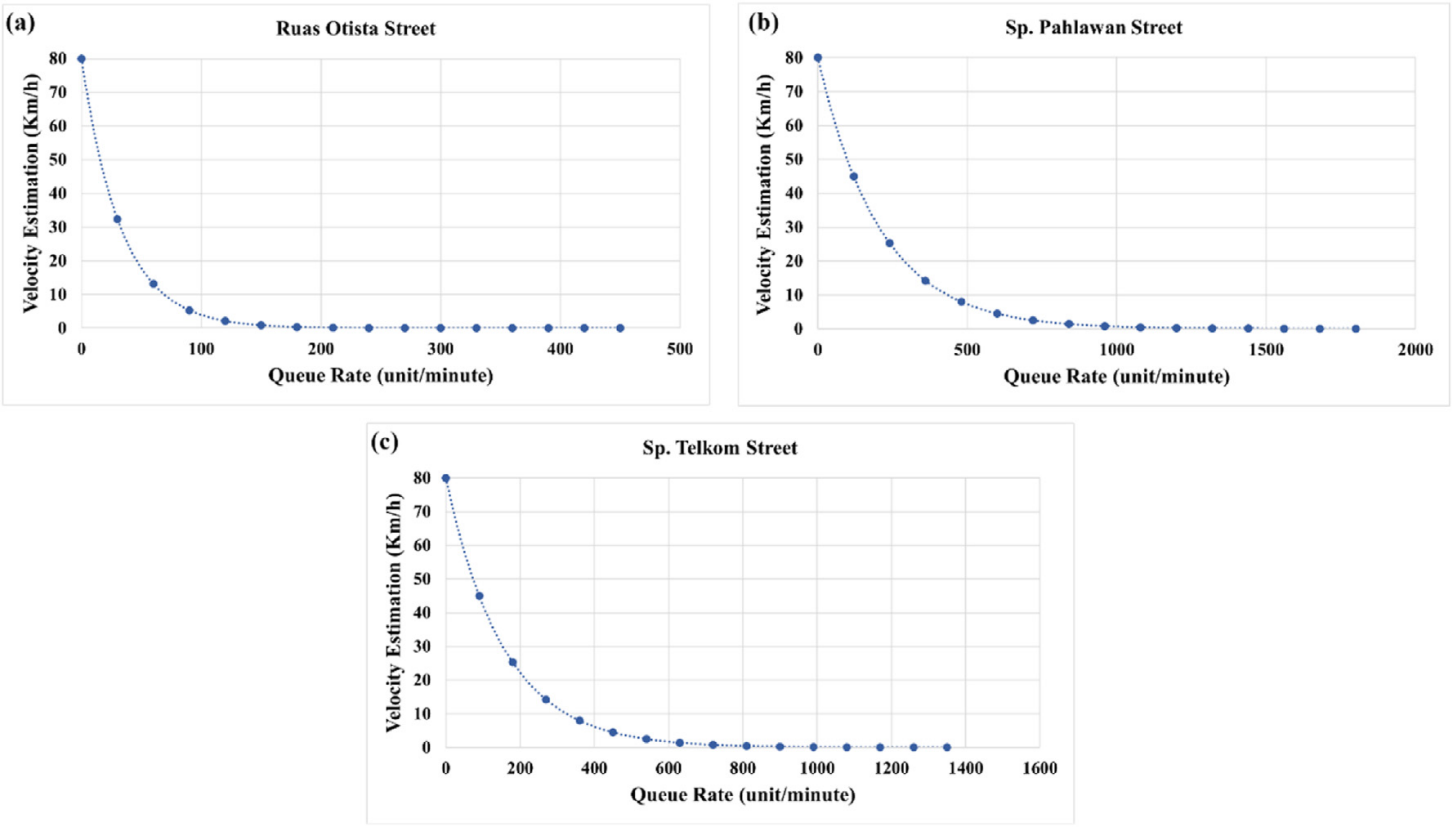
Vehicle velocity estimation at (a) Urban I, (b) Urban III, and (c) Urban IV.

The assumption at the initial time $\left(t_{0}=0\right)$ the vehicle is on the bottom line (bottom frame) of Figure 7, the initial speed of the vehicle is $v_{0}=80\;km/hour$ with the assumption that it is free of obstacles (λ = 0). Based on the data obtained from Urban I by measuring the length of the street (x = 18m) which is in picture frame Figure 13a when the condition is 0<λ<30, the vehicle travel time as far as x is 2 s. So, the velocity at condition (0<λ<30) is 9 m/s which is equivalent to 34.4 km/h. By using Eq. (5), the constant value (*k*) will be obtained for a one-way type of street $k=0,03013\;{unit}^{-1}\text{.}$ Based on the data obtained from Urban III by measuring the length of the road (*x* = 25) which in Figure 13b when condition is 0<λ<120, the vehicle travels as far as *x* is 2 s. So, the speed at condition (0<λ<120) is 12,5 m/s which is equivalent to 45 km/h. By using Eq. (5), the constant value (*k*) will be obtained for streets that have four intersections is $k=0,0048\;{unit}^{-1}$. While Urban IV is measured by measuring the length of road (*x* = 25) which in Figure 13c when condition is 0<λ<90, the vehicle travels as far as *x* is 2 s. So, the speed at condition (0<λ<90) is 12,5 m/s which is equivalent to 45 km/h. By using Eq. (5), the constant value (*k*) will be obtained for a street which has three intersections is $k=0,0064\;{unit}^{-1}$. The correlation of vehicle velocity with the queue rate (*λ*) is inversely exponential. The higher the queue rate (*λ*), the lower the speed of vehicles traveling on the road. That statement is supported by the measurement data in the following figure: Urban I (Figure 14a), Urban III (Figure 14b), and Urban IV (Figure 14c).

### 4.4 Result of anthropogenic heat flux analysis

We have calculated the anthropogenic heat flux caused by the internal combustion vehicle using Eq. (8). The heat energy (Qv) emitted from the combustion of vehicle fuel depends on the number of vehicles, type of fuel, heat contribution area, duration of congestion, and long traffic jams. In the calculation two types of vehicles were used, namely motorcycles and cars. As for the type of fuel, most vehicles use the type of fuel with RON 90–92 with a net heat combustion (NHC) of 47873 kJ/kg. Average fuel economy (FEij) for motorcycles are 40890 m/liter and cars are 5240 m/liter. By applying Eq. (9), we get the energy (EV) used by the motorcycle with an interval of 798–837 J/m and car with an interval of 5033–6532 J/m. Therefore that the estimated heat energy (QV) of vehicles that flows every hour can be seen in Table 4 below.

**Table 4:** Heat flux effect of queue rate in Urban I Bandung area on October 10th, 2021

Time [hh:mm]	Motor-cycles [unit]	Cars [unit]	Area [m^2^] x 10^6^	Queue Length (L) [m]	Ev Motorcycle [J/m]	Ev Car [J/m]	Qv Motorcycles [W/m^2^]	Qv Cars [W/m^2^]	Heat Coefficient ($\bar{h}$) [W/m^2^C]	Total Qv vehicle [W/m^2^]	${UHI}_{T}$ [C]
00:00	0	15	167,3	3,11	837,1	6532,28	0,00	0,00	3,09	0,00	0,00
01:00	0	11	167,3	3,08	837,1	6532,28	0,00	0,00	3,11	0,00	0,00
02:00	0	0	167,3	3,00	837,1	6532,28	0,00	0,00	3,15	0,00	0,00
03:00	0	8	167,3	3,06	837,1	6532,28	0,00	0,00	3,12	0,00	0,00
04:00	0	19	167,3	3,14	837,1	6532,28	0,00	0,00	3,08	0,00	0,00
05:00	51	220	167,3	4,91	837,1	6532,28	0,00	0,04	2,46	0,04	0,02
06:00	45	428	167,3	6,17	837,1	6532,28	0,00	0,10	2,20	0,10	0,05
07:00	148	775	167,3	8,56	837,1	6532,28	0,01	0,26	1,86	0,27	0,14
08:00	343	1288	167,3	11,39	837,1	6532,28	0,02	0,57	1,62	0,59	0,37
09:00	507	1554	167,3	12,67	837,1	6532,28	0,03	0,77	1,53	0,80	0,52
10:00	422	1925	167,3	13,38	837,1	6532,28	0,03	1,01	1,49	1,03	0,69
11:00	298	2125	167,3	13,56	837,1	6532,28	0,02	1,13	1,48	1,15	0,77
12:00	725	2400	167,3	14,88	837,1	6532,28	0,05	1,39	1,41	1,45	1,03
13:00	2021	4788	167,3	17,51	837,1	6532,28	0,18	3,27	1,30	3,45	2,65
14:00	1605	9158	167,3	17,93	837,1	6532,28	0,14	6,41	1,29	6,56	5,08
15:00	1323	10537	167,3	17,96	837,1	6532,28	0,12	7,39	1,29	7,51	5,82
16:00	1764	2514	167,3	16,25	837,1	6532,28	0,14	1,60	1,35	1,74	1,29
17:00	1145	2433	167,3	15,51	837,1	6532,28	0,09	1,47	1,39	1,56	1,12
18:00	7	3071	167,3	14,80	837,1	6532,28	0,00	1,77	1,42	1,78	1,25
19:00	0	3189	167,3	14,98	837,1	6532,28	0,00	1,87	1,41	1,87	1,32
20:00	2	163	167,3	4,19	837,1	6532,28	0,00	0,03	2,66	0,03	0,01
21:00	0	84	167,3	3,62	837,1	6532,28	0,00	0,01	2,87	0,01	0,00

Based on the heat emitted by internal combustion (Qv) obtained from the calculation, then we calculate the temperature increase caused by Qv with Eq. (10) which depends on the QV data and heat transfer coefficient ($\bar{h}$). The value of heat transfer coefficient ($\bar{h}$) is obtained from environmental temperature conditions (temperature film), traffic queue length, Reynolds constant, and Prandtl number. Approach to environmental conditions with the assumption of area coverage for heat energy that spreads with the approximation value of the area of the city of Bandung. Therefore, we get the heat transfer coefficient ($\bar{h}$) as shown in the table below. The results of the Realtime UHI Theoretic (${UHI}_{T}^{RT}$) calculation (see Table 4) generated as a result of heat emitted by internal combustion (Qv) show that the effect of the queue rate value is directly proportional. The more congested traffic condition (large queue rate), the UHI _T_ will increase.

In general, the summary of correlation data for queue rate, traffic jams, weather conditions, urban temperature, suburban temperature, and UHI temperature is shown in Table 5. Based on that table we describe Vehicle Queue Rate (unit/minute) as (λ), Estimation velocity of vehicle (km/hour) as ($\bar{v}$), Minimum temperature on suburban area (°C) (${T1}_{\min}$ s), Minimum temperature on urban area (°C) as (${T2}_{\min})$, Maximum temperature on suburban area (°C) (${T1}_{\max}$), Maximum temperature on urban area (°C) as (${T2}_{\max}$), Average temperature on suburban area (°C) as (${\bar{T1}}_{ave}$), Average temperature on urban area (°C) as (${\bar{T2}}_{ave}$), and Temperature of Urban Heat Island Phenomenon based on Aggregate Measurement (${UHI}_{M}^{Ag})$ and Aggregate Theoretic (${UHI}_{T}^{Ag}$) (°C).

**Table 5:** Correlation of Traffic condition, weather, temperature, ${UHI}_{M}$, and ${UHI}_{T}$

No.	Date	Session	Weather	Traffic	λ	$\bar{v}$	${T1}_{\max}$	${T2}_{\max}$	${T1}_{avg}$	${T2}_{avg}$	${UHI}_{M}$	${UHI}_{T}$
					unit/minute	(km/h)	(°C)	(°C)	(°C)	(°C)	(°C)	(°C)
Urban I												
1	8/10 2021	05:00–11:59	Cloudy sunny	Smooth	70	10	32,79	34,31	26,95	29,11	1,52	1,95
		12:00–18:00	Rainstorm	Jam	174	0	31,38	32,31	27,08	27,86	0,93	5,66
2	10/10 2021	05:00–11:59	Cloudy sunny	Smooth	24	39	34,05	36,25	27,14	29,12	2,20	0,36
		12:00–18:00	Cloudy sunny	Jam	112	3	34,73	38,42	31,50	32,56	3,69	3,53
3	11/10 2021	05:00–11:59	Cloudy sunny	Smooth	73	9	32,04	35,98	27,17	29,91	1,20	2,07
		12:00–18:00	Cloudy sunny	Smooth	63	12	32,62	36,94	28,34	30,34	2,32	1,70
Urban III												
4	31/10 2021	05:00–11:59	Cloudy sunny	Jam	518	7	30,18	36,08	26,12	29,58	5,90	3,82
		12:00–18:00	Light rain	Jam	713	3	29,43	32,31	23,83	25,24	2,88	5,62
5	03/11 2021	05:00–11:59	sunny	Jam	860	1	29,76	35,44	24,42	28,22	5,68	6,93
		12:00–18:00	Cloudy sunny	Jam	676	3	29,77	34,52	24,55	25,72	4,75	5,26
6	05/11 2021	05:00–11:59	Cloudy sunny	Jam	820	2	28,71	34,34	23,17	25,33	5,63	6,54
		12:00–18:00	Cloudy sunny	Jam	613	4	31,17	38,64	28,19	32,17	7,47	4,68
Urban IV												
7	27/10 2021	05:00–11:59	Cloudy Sunny	Smooth	133	34	30,23	31,24	25,77	26,81	1,01	0,75
		12:00–18:00	Rainstorm	Smooth	141	32	30,11	30,84	25,82	26,50	0,73	0,83
8	28/10 2021	05:00–11:59	Cloudy	Jam	753	1	27,66	28,90	23,32	25,05	1,24	8,15
		12:00–18:00	Light Rain	Jam	715	1	27,24	29,23	25,52	26,62	1,99	7,73
9	29/10 2021	05:00–11:59	Cloudy	Smooth	331	10	29,92	31,72	24,72	26,17	1,80	3,08
		12:00–18:00	Light Rain	Smooth	258	15	28,13	28,55	22,96	24,22	0,42	2,18

The phenomenon of temperature in urban areas as mentioned in Table 5 tends to have higher temperatures than in suburban areas. Many factors influence it. One of the focuses of this research is to observe the increase in temperature at the level of the number of passing vehicles, mentioned as the queue rate (*λ*). In this paper, it is explained that traffic jam can be determined based on the vehicle velocity (*v*), the number of vehicle passing every minute based on the queue rate (*λ*), and the UHI Measurement temperature, which is known from the difference between urban and suburban temperature. The extreme changing microclimate and UHI are influenced by queue rate (*λ*), weather conditions, and other factor as our limitation such a human metabolism and energy consumption of building. The results ${UHI}_{T}^{Ag}$ calculation (see Table 5) is highly correlated with an increase in ${UHI}_{M}^{Ag}$ temperature based on measurements by sensors in sunny weather. This means that the effects of congestion and queue rate provide a large increase in temperature in the urban area.

Based on Table 5, it displayed that the air temperature is $T_{urban\;III}>T_{urban\;IV}>T_{urban\;II}>T_{urban\;I\;.}$ Hence, it is also comparable to the difference in UHI. In other word, the density of vehicle passing in the urban area is $\lambda_{urban\;III}>\;\lambda_{urban\;IV}>\;\lambda_{urban\;II}>\;\lambda_{urban\;I}$.

**Figure 15:**
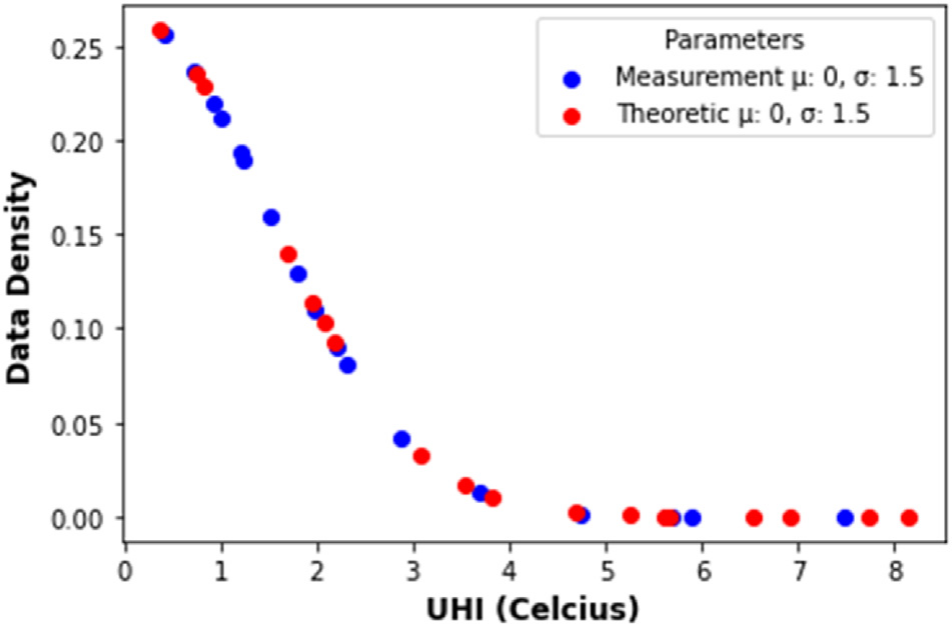
Data normality test of aggregated UHI measurement (${UHI}_{M}^{Ag})$ and theoretics (${UHI}_{T}^{Ag})$

In order to show effectiveness of aggregate UHI Theoretics (${UHI}_{T}^{Ag}$) in comparison to the existing UHI Measurements (${UHI}_{M}^{Ag}$). First the data density plot of ${UHI}_{T}^{Ag}$ and ${UHI}_{M}^{Ag}$ took place to see the difference between two measures (see Figure 15). Data density (Figure 15) that means density probability function of normal distribution. To clearly see the difference between two measures, a statistical approach is applied. One of the approaches in statistics to deal with comparing two samples of continuous data is Kolmogorov Smirnov (K–S Test). K–S Test is used to analyze whether the sample comes from a population with a specific distribution (Sahoo, 2013) (Yahya, 2017).

The test statistic, p−value, should be compared with the critical value (α) where α=5%. The null hypothesis was that ${UHI}_{M}^{Ag}$ and ${UHI}_{T}^{Ag}$ came from same distribution. The chance of rejecting the null hypothesis when p−value is less than 5% regardless of samples size. In this testing *p-value* is 0.5025, therefore the ${UHI}_{M}^{Ag}$ and ${UHI}_{T}^{Ag}$ came from the same distribution.

### 4.5 Result of Microclimate analysis of urban heat island (UHI)

**Figure 16:**
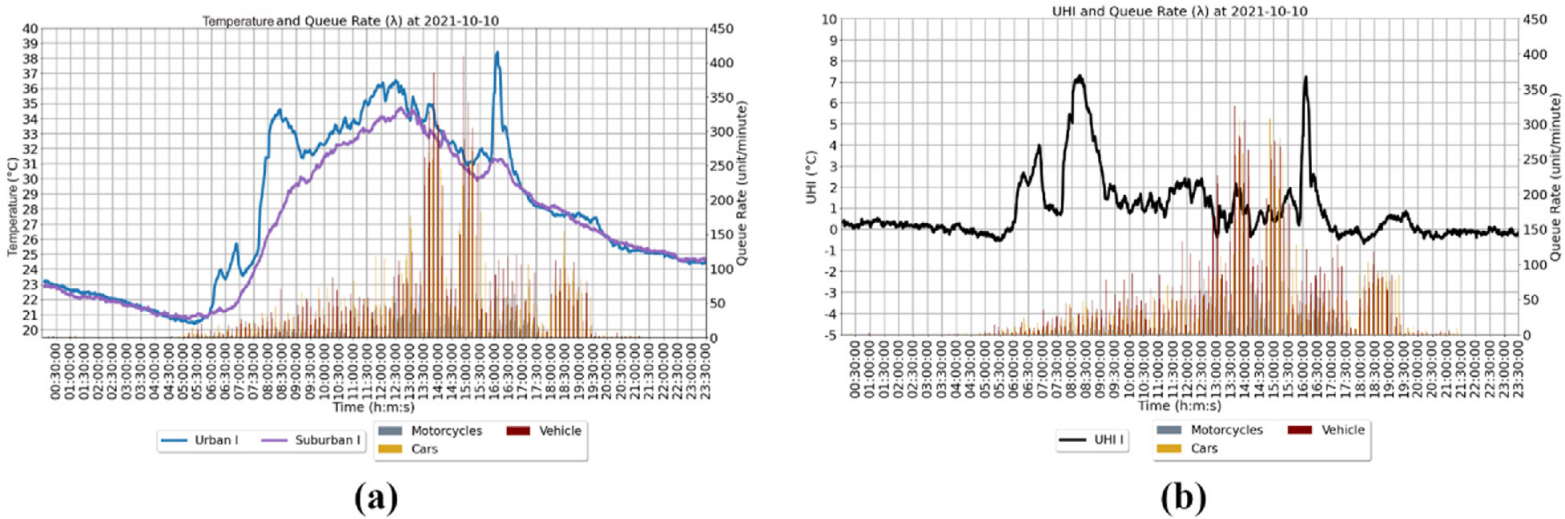
(a) Temperature and queue rate graph, (b) UHI and queue rate graph.

Throughout the observation, it was found that the duration of traffic jams and queue rate (λ) significantly affected the increase in urban air temperature. As shown in Figure 16 a, the duration of the traffic jam starts from 13:00 to 15:30 with λ = 350 units/minute with sunny weather conditions, and the urban temperature reaches 38.61 Celsius. Based on these conditions, the real time UHI temperature (${UHI}_{M}^{RT}$) is obtained based on Urban I data on October 10, 2021, which is 7 Celsius (Figure 16 b). Therefore, the longer the duration of the traffic jam, the more significant the temperature increases. Based on real condition of observation that there is a time lag between the queue rate (λ) of passing vehicles and the spread of heat that occurs as a cause of extreme air temperature in urban areas. In addition, the rainy weather factor significantly affects the decrease in urban temperature.

**Figure 17:**
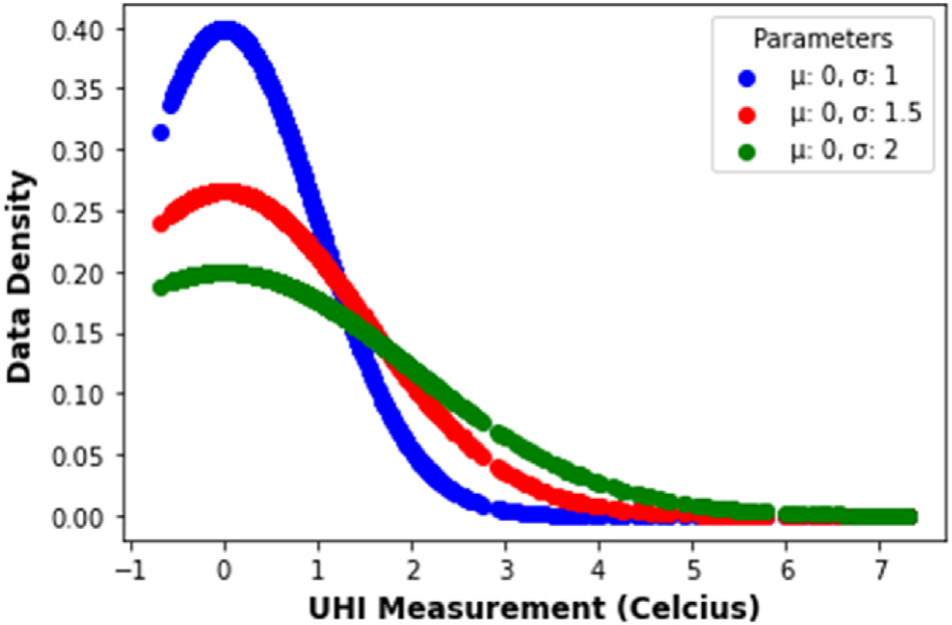
Pdf of normal distribution real time UHI measurement (${UHI}_{M}^{RT}$).

A normal distribution with μ=0 and σ=1, that is X∼N(0,1), is called the standard normal distrbution. The probability of density function (pdf) $f_{X}\left(x\right)$ was applied on this visual distibution test. The plot of pdf for different values of μ and σ, are provide in Figure 17, respectively by using scipy library in python. It is clear form (Figure 17) that the graphs of the pdf $f_{X}\left(x\right)$ of UHI variable, X∼N(μ,σ), is not symetric about mean, μ, that $f_{x}\left(\mu +x\right)\neq$ $f_{x}\left(\mu -x\right)$ (Ahsanullah et al., 2014). Therefore this ${UHI}_{M}^{RT}$ was not normal distributed.

To clearly see whether ${UHI}_{M}^{RT}$ data was not normally distributed, a statistical approach is applied. One of the approaches in statistics to deal with data normality is Kolmogorov Smirnov (K–S Test). The test statistic, p−value, should be compared with the critical value (α) where α=5%. The null hypothesis was that ${UHI}_{M}^{RT}$ data normal distributed. The chance of rejecting the null hypothesis when p−value is less than 5% regardless of samples size. In this testing *p-value* is 3.0389e-130, therefore with sufficient evidence the ${UHI}_{M}^{RT}$ data not come from a normal distribution.

**Figure 18:**
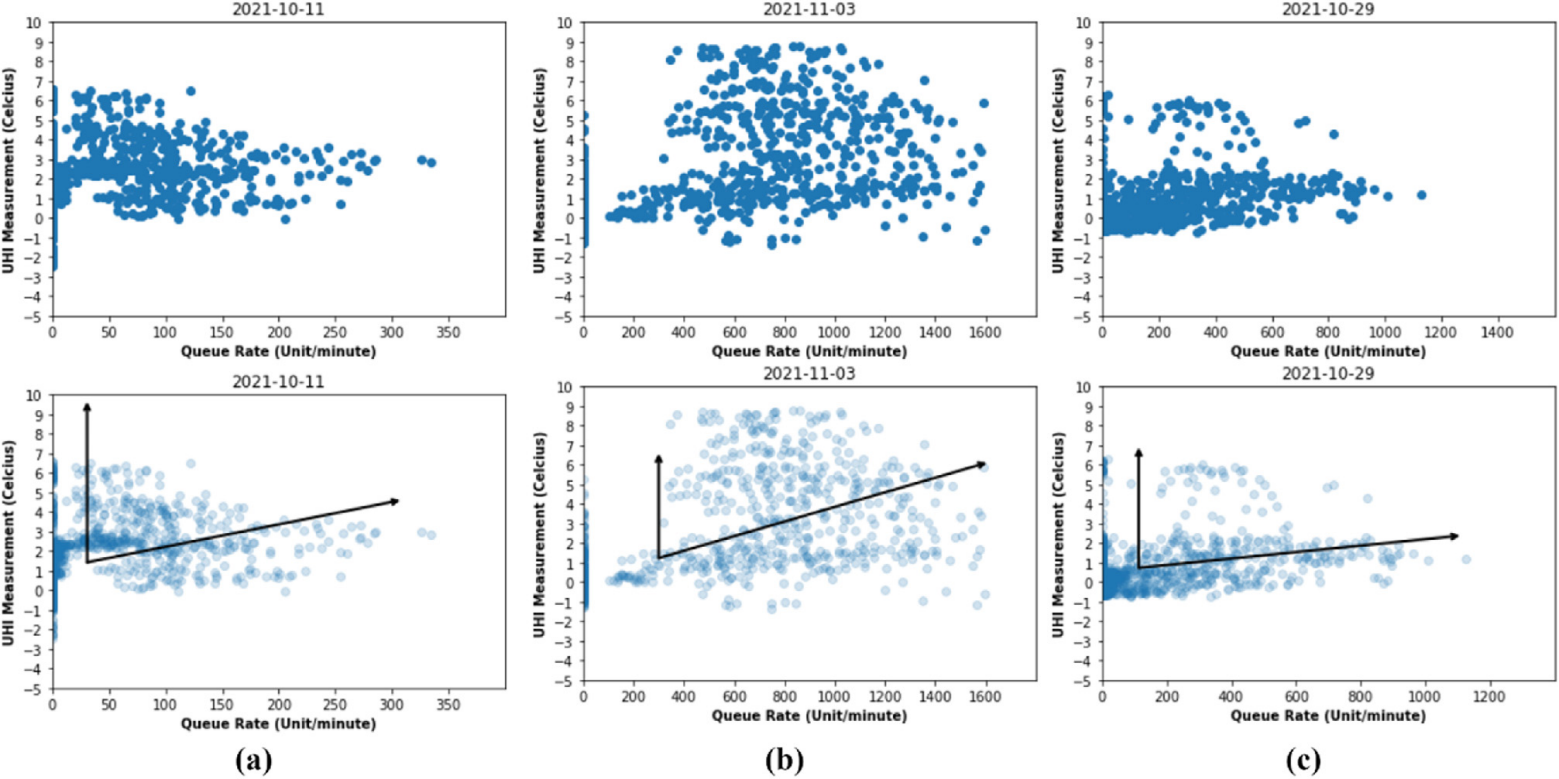
PC1 and PC2 of sample data on (a) Urban I, (b) Urban III, and (c) Urban IV.

After the normality test is completed, it is continued with Principal Component Analysis (PCA), Principal Component Regression (PCR), and non-parametric data analysis such as Spearman correlation. In the principal component analysis process, we apply using 2-dimensional data consisting of ${UHI}_{M}^{RT}$ and queue rate for 24 h. The results of the analysis show that there are two trends, they are a positive trend and no correlation as shown in measurement data Urban I (Figure 18 a), Urban III (Figure 18 b) and Urban IV (Figure 18 c). The first trend is the positive trend in the vector indicating a correlation between the increase in UHI temperature due to queue rate. Meanwhile, the second trend vector shows that there is no correlation between the data on the increase in ${UHI}_{M}^{RT}$ due to queue rate = 0. We have done a comprehensive analysis of the first trend using principal component regression, that based on the results of hypothesis testing there is a strong influence of increasing ${UHI}_{M}^{RT}$ temperature due to queue rate. However, in the second trend, there is no correlation between the increase in ${UHI}_{M}^{RT}$ temperature and queue rate due to the relative absence of vehicles passing by at midnight between 10.00 pm - 05.00 am. In our limitation of observation, there are differences in building energy consumption between urban and suburban environments which result in changes in UHI.

Based on the visualization, it is obvious that there is a nearly linear relationship between the ${UHI}_{M}^{RT}$ and queue rate variables. This is reminiscent of the linear regression data we explored in Linear Regression, but the problem setting here is slightly different: rather than attempting to predict the UHI values from the queue rate, the unsupervised learning problem attempts to learn about the relationship between the ${UHI}_{M}^{RT}$ and queue rate values. In principal component analysis, this relationship is quantified by finding a list of the principal component axes (PC-1, PC-2, … PC-n) in the data and using those axes to describe the dataset.

**Figure 19:**
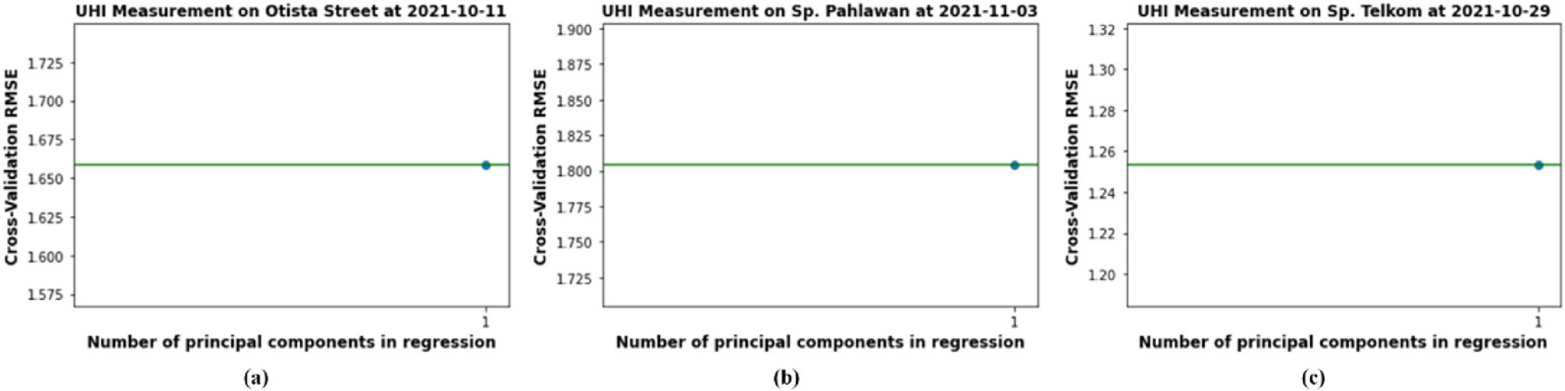
Number of principal components (a) urban i, (b) urban III, (c) urban IV.

In ${UHI}_{M}^{RT}$ data, there is only one independent variable. Meanwhile, in the ${UHI}_{M}^{Ag}$ data, there are two independent variables. The independent variables in real time observation use queue rate data, while in aggregate observation use queue rates and weather condition. Therefore, that by applying PCR, only one principal component (PC) is obtained in real-time data observation as shown in the graph of the analysis data in the Urban I (Figure 19 a), Urban III (Figure 19 b) and Urban IV (Figure 19 c).

**Figure 20:**
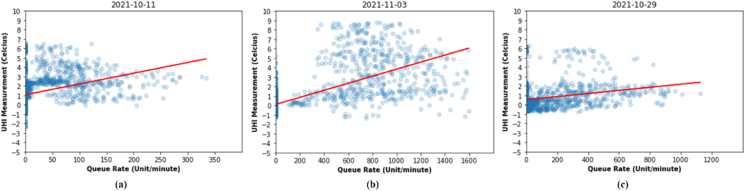
PCR (a) urban i, (b) urban III, and (c) urban IV.

The results obtained from the PCR show that PCR model give the same results as unregularization linear regression. For the purposes of predicting UHI by using ${UHI}_{T}^{Ag}$, the RMSE (see Table 6) results given by Urban III (Figure 20 b) are slightly worse than Urban I (Figure 20 a) and Urban IV (Figure 20 c). However, this is a real condition of the ${UHI}_{M}^{RT}$ process. Urban III gives better results in terms of seeing the correlation between ${UHI}_{M}^{RT}$ and queue rate (Figure 20 b). The results of the ${UHI}_{M}^{RT}$ in each urban location are due to the influence of weather condition from the ${UHI}_{M}^{RT}$ process. Judging from the weather conditions at each measurement time of each urban area, urban I is cloudy, urban III is sunny, and urban IV is rainy.

**Table 6:** RMSE Train Set and RMSE Test Set of ${UHI}_{M}^{RT}$ and queue rate data correlation.

Parameters	RMSE (Train Set)			RMSE (Test Set)		
Model	Urban I	Urban III	Urban IV	Urban I	Urban III	Urban IV
Linear Regression	1,65	1,80	1,25	1,57	1,71	1,12
PCR (1 component)	1,65	1,80	1,25	1,57	1,71	1,12

**Figure 21:**
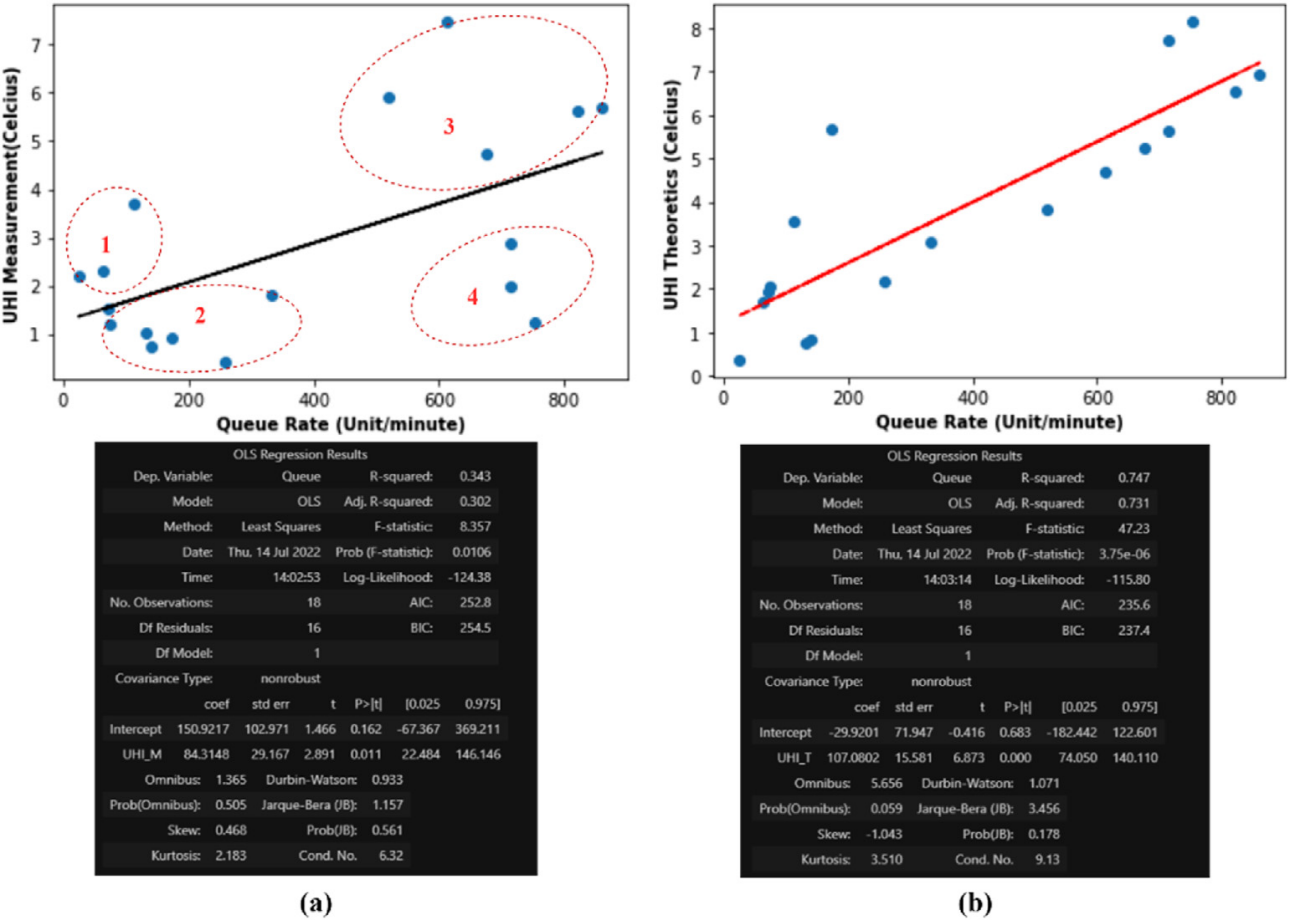
Regression and statistics summary of aggregated data (a) UHI measurement (${UHI}_{M}^{Ag})$, (b) UHI theoretics (${UHI}_{T}^{Ag})$

In the data sample with varying weather conditions and the queue rate as independent variables in each session as shown in Table 5. We performed regression analysis to test the hypothesis null (*H0*) and hypothesis alternative (*H1*), whether queue rate affects the increase in UHI temperature. The assumption used is that the accompanying variables/covariates (weather) are fixed, measured without error, and not correlated with the treatment (queue rate) being tested. Based on hypothesis testing with analysis of variance, the p-value is 4.71e-09 indicates that the p-value is smaller than the alpha value = 0.05. This means that the *H0* can be rejected. Therefore, it can be concluded that *H1* can be accepted that satisfied if the queue rate influences changes in UHI. From the results of the analysis of regressions as presented in Figure 21 below, it is obtained that $R_{M}^{2}=0,343$ of ${UHI}_{M}^{Ag}$ (Figure 21 a) and $R_{T}^{2}=0,747$ of ${UHI}_{T}^{Ag}$ (Figure 21b). The value of R-square obtained from $R_{M}^{2}$ and $R_{T}^{2}$ is a sample estimate which quantifies how much the dependent variable is determine by the independent variables, in term of proportion of variance (Chicco et al., 2021). Although $R_{M}^{2}$ is smaller than $R_{T}^{2}$, R square 0,343 is an R-square category with a large effect (Frey, 2022). Therefore, it can be concluded that there is an effect of queue rate on the increase in UHI. Thus, after controlling for queue rate, anthropogenic heat flux factors caused by population density, human metabolism, and weather factors still affect changes in UHI temperature. The covariance value in the calculation of 2-dimensional data (${UHI}_{M}^{Ag}$ of queue rate) and data (${UHI}_{T}^{Ag}$ of queue rate) shows a positive trend with a value of 364,43 and 624,73, which means that the higher the queue rate, the UHI will increase. Based on Spearman correlation test, the result show that the spearman rank correlation ${UHI}_{M}^{Ag}=0,395$ and ${UHI}_{T}^{Ag}=0,872$, and the corresponding p-value ${UHI}_{M}^{Ag}$ = 0,104 and ${UHI}_{T}^{Ag}$ = 2,401e-06.

The resulting covariance is quite large in the queue rate data because we used samples in three different locations as shown in Table 5. From a fitting model, the relationship between UHI and queue rate shows a positive trend. However, in this sample data, we have to consider accompanying factors as our limitation such as the influence building energy consumption, and human metabolism when measuring temperature in urban and suburban environments. Referring to Figure 21 a, there are four data clusters caused by differences in weather when measuring UHI temperatures. Clusters 1 and 3 data were measured when the weather was sunny and cloudy. while clusters 2 and 4 data were measured when the weather was light rain and rainstorm.

## 5 Conclusion and future work

This study aimed to investigate microclimate analysis by looking into the UHI and other aspects (e.g., queue rate, weather) that impact on the environment. To measure the UHI, we leveraged IoT, which is based on the fix stations, to collect the temperature data. The data were collected for every minute and were utilized using the cloud service prior to the analysis and visualization. The system can be regarded as a hyperlocal low energy consumption fixed station sensor integrated with IoT to measure the air temperature that can last for about three (3) months. For the integration with the anthropogenic heat flux analysis, we perform vehicle detection system to estimate the number of vehicles that pass every minute based on ATCS's CCTV. Since the temperature data collection using IoT is a tedious job, this study proposed a theoretical UHI as an alternative of UHI measurement. The theoretical UHI, which is a derivation from anthropogenic heat flux, was statistically tested with UHI Measurement. The result showed that the two measurements significantly having similar distribution.

The result showed that the daily real time UHI can reach 7 °C in the rush hour, which caused traffic congestion. It proved that the air temperature in urban areas with dense vehicle traffic has a higher average air temperature than in suburban areas. In correlation with weather, traffic jams occur and UHI increases significantly when the weather is sunny. On the other hand, the traffic flows and the UHI did not increase significantly when the weather is rainy. The increase in temperature in the urban environment caused by traffic jams is directly proportional to the internal exhaust emissions. Exhaust gas emissions such as carbon monoxide (CO), nitrogen oxides (NO), hydrocarbon (HC), carbon dioxide (CO_2_), sulfur oxides (SO_2_) and particulate matter (PM_10_) in an urban environment with traffic jams will increase the entropy of the environment so that the air in urban areas tends to be not fresh and hotter than the suburbs where there are no vehicles activity. However, during rainy times, the humidity of the surrounding air reduces the entropy of the environment because exhaust gas emissions tend to be bound by water particles (H_2_O). Based on the analysis using the correlation between UHI and queue rate, the correlation is quite large, with the R-squared value of $R_{M}^{2}=0,343$ and $R_{T}^{2}=0,747$. The fixed station measurements provide the potential for better UHI observation because it can represent temperatures in the microclimate and provide a fine granularity in terms of time.

Although the result showed the relationship between UHI and queue rate, there are some limitations on this work. First, the power consumption is limited for the fix station measurement so that intermittent data cause some missing values. Second, the vehicle detection system is developed as a standalone program with no real time configuration. It is expected to expand the implementation with a web application and utilize the data for predictive analytics using machine learning approaches.

## Declarations

### Author contribution statement

Emir Husni, Galang Adira Prayoga, Josua Dion Tamba, Yulia Retnowati, Fachri.

Imam Fauzandi, Rahadian Yusuf, Bernardo Nugroho Yahya: Conceived and designed the experiment.

Emir Husni, Galang Adira Prayoga, Josua Dion Tamba, Yulia Retnowati, Fachri.

Imam Fauzandi: Performed the experiments.

Emir Husni, Galang Adira Prayoga, Rahadian Yusuf, Bernardo Nugroho Yahya:

Analyzed and interpreted the data.

Emir Husni, Galang Adira Prayoga, Yulia Retnowati, Rahadian Yusuf, Bernardo.

Nugroho Yahya: Contributed reagents, materials, analysis tools or data.

Emir Husni, Galang Adira Prayoga, Josua Dion Tamba, Yulia Retnowati, Fachri.

Imam Fauzandi, Rahadian Yusuf, Bernardo Nugroho Yahya: Wrote the paper.

### Funding statement

Emir Husni was supported by Institut Teknologi Bandung.

### Data availability statement

Data will be made available on request.

### Declaration of interest's statement

The authors declare no conflict of interest.

### Additional information

No additional information is available for this paper.
